# Luminescent sensing of conformational integrin activation in living cells

**DOI:** 10.1016/j.celrep.2025.115319

**Published:** 2025-02-17

**Authors:** Giulia Villari, Noemi Gioelli, Marta Gino, Heng Zhang, Kelly Hodge, Francesca Cordero, Sara Zanivan, Jieqing Zhu, Guido Serini

**Affiliations:** 1 Department of Oncology, University of Torino School of Medicine, Candiolo, TO, Italy; 2 Candiolo Cancer Institute - Fondazione del Piemonte per l’Oncologia (FPO) Istituto di Ricovero e Cura a Carattere Scientifico (IRCCS), Candiolo, TO, Italy; 3 Thrombosis and Hemostasis Program, Versiti Blood Research Institute, Milwaukee, WI, USA; 4 Cancer Research UK Scotland Institute, Glasgow, UK; 5 Department of Computer Science, University of Torino, Torino, Italy; 6 School of Cancer Sciences, University of Glasgow, Glasgow, UK; 7 Department of Biochemistry, Medical College of Wisconsin, Milwaukee, WI, USA

**Keywords:** integrin activation, luminescence, conformational sensor, integrin inhibitors, endothelial cells, angiogenesis

## Abstract

Integrins are major receptors for secreted extracellular matrix, playing crucial roles in physiological and pathological contexts, such as angiogenesis and cancer. Regulation of the transition between inactive and active conformation is key for integrins to fulfill their functions, and pharmacological control of those dynamics may have therapeutic applications. We create and validate a prototypic luminescent β1 integrin activation sensor (β1IAS) by introducing a split luciferase into an activation reporting site between the βI and the hybrid domains. As a recombinant protein in both solution and living cells, β1IAS accurately reports β1 integrin activation in response to (bio)chemical and physical stimuli. A short interfering RNA (siRNA) high-throughput screening on live β1IAS knockin endothelial cells unveils hitherto unknown regulators of β1 integrin activation, such as β1 integrin inhibitors E3 ligase Pja2 and vascular endothelial growth factor B (VEGF-B). This split-luciferase-based strategy provides an *in situ* label-free measurement of integrin activation and may be applicable to other β integrins and receptors.

## Highlights

• β1IAS reports β1 integrin activation in response to biochemical and physical stimuli
• siRNA HTS on β1IAS KI endothelial cells unveils unknown regulators of β1 integrin activation
• E3 ubiquitin ligase Pja2 promotes kindlin-2 degradation and inhibits β1 integrin activation
• VEGF-B is a so far unrecognized effective inhibitor of β1 integrin activation

## Graphical abstract

**Figure:**
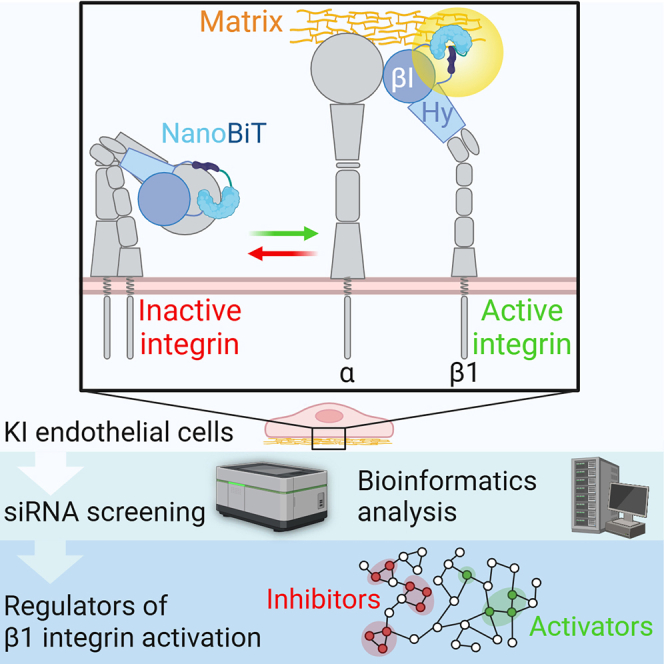

## Teaser

Villari et al. present the generation of a luminescent reporter for a β1 integrin activation sensor (β1IAS). A siRNA HTS on β1IAS KI endothelial cells revealed previously uncharacterized regulators of β1 integrin activation: the E3 ubiquitin ligase Pja2, which promotes kindlin-2 degradation, and the VEGF-B, which stabilizes β1 integrin inactive conformation.

## Introduction

Integrins are the main adhesion receptors through which cells of metazoans physically connect their actin cytoskeleton to polymeric extracellular matrix (ECM) ligands.^1^ These heterodimeric αβ transmembrane receptors control essential physiological functions, such as organogenesis,^2^ angiogenesis,^3^ neuronal connectivity,^4^ platelet aggregation,^5^ and leukocyte extravasation.^6^ On the other hand, alterations in integrin-mediated adhesion are involved in the pathogenesis of several diseases, such as coagulopathies,^5^ immune disorders,^7^ and cancer.^8^ The core of the regulation of integrin adhesive functions lies in the spatiotemporal modulation of their large-scale conformational changes within the extracellular domains composed of headpiece and leg portions.^9^^,^^10^ On the cell surface, integrins exist in a dynamic equilibrium between inactive (bent-closed and low-affinity) and active (extended-open and high-affinity) conformations,^9^ which is regulated by signal transduction pathways that may have been incompletely discovered and whose targeting may provide opportunities for therapeutic intervention.^11^ Thus, it is crucial to devise methods that allow a simple, quantitative, and noninvasive measurement of integrin conformational states in live cells and may be exploited in high-throughput screenings (HTSs) to identify until-now unnoticed ligands, receptors, biochemical signaling pathways, or drugs capable of inhibiting or activating integrin function. Here, we describe the development of a bioorthogonal luminescent sensor that, when knocked in at a critical position within the human β1 integrin gene (*ITGB1*), allows a quantitative measurement of the conformational transition happening during β1 integrin activation in living vascular endothelial cells (ECs) and the identification of so far unrecognized β1 integrin regulators by means of a short interfering RNA (siRNA) HTS.

## Results

### Design and characterization of a luminescent β1 integrin activation sensor in living cells

In inactive integrins, the α and β subunits are tightly associated along their whole length, and the extracellular N-terminal headpiece, which displays a closed, low-affinity conformation, is bent over the plasma membrane proximal leg.^12^^,^^13^ In active integrins, while the headpiece moves away from the leg, adopting an open, high-affinity conformation, the fully extended α and β subunits separate, remaining in contact only at the ligand binding site.^12^^,^^13^ Following the split of the two subunits, the cytosolic domain of the β chain is then free to associate with the talin and kindlin adaptors that connect integrins to the actin cytoskeleton, thus enabling ECM adhesion.^9^

**Figure 1:**
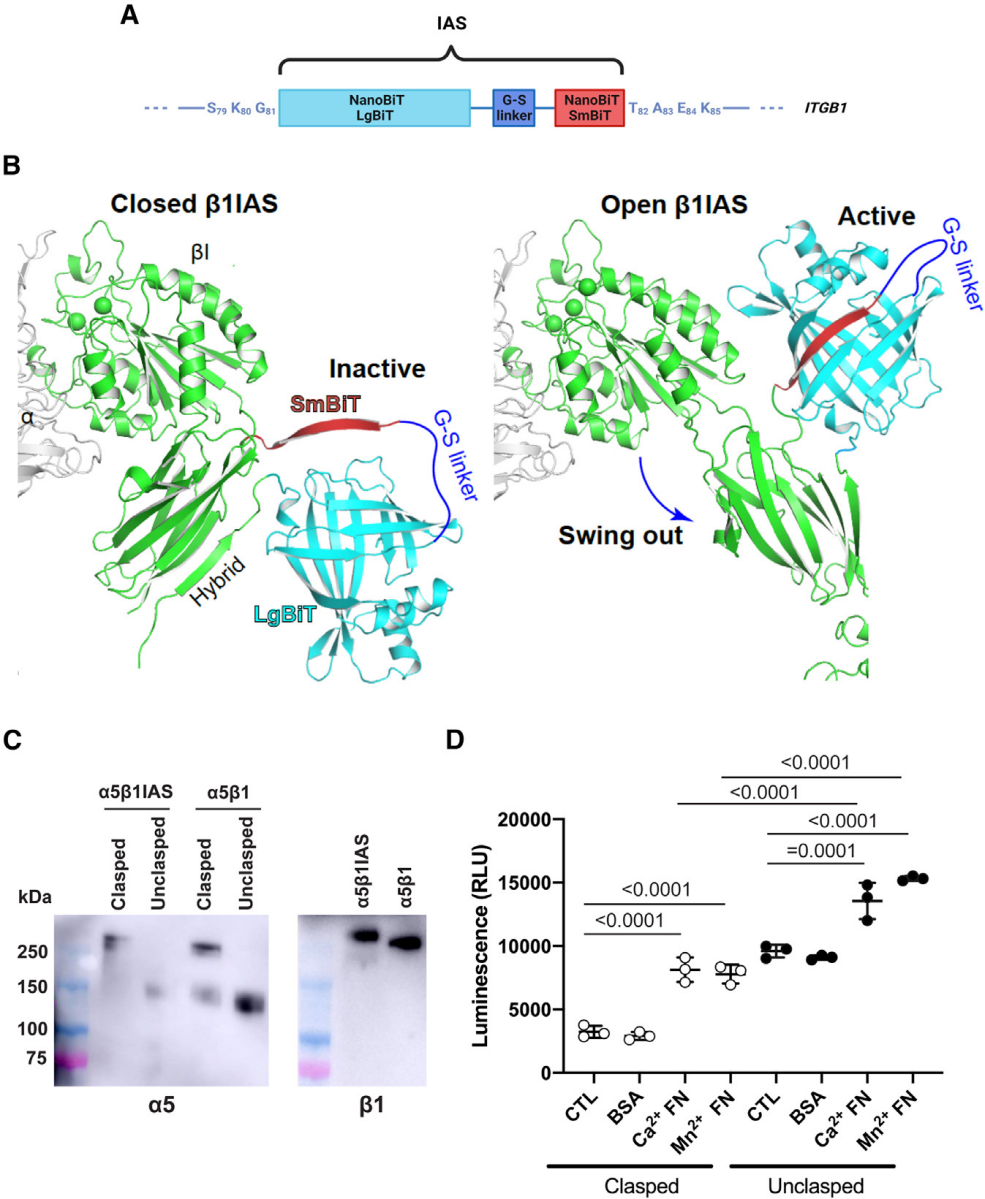
Design and validation of β1IAS purified extracellular protein (A) Diagram of β1 integrin activation sensor (β1IAS) construct. Created with BioRender.com. (B) Molecular modeling of NanoBiT activity reconstruction in α5β1IAS during hybrid domain swing-out and headpiece opening. The headpiece structures of β1 integrin in closed and open conformations were modeled from PDB: 7CEB and 7CEB. The structure of NanoBiT was modeled from PDB: 7SNX. (C) The ectodomains of α5β1 WT and α5β1IAS were expressed with a C-terminal disulfide-linked ACID-BASE coiled coil in Expi293F or HEK293 cells. The presence of purified soluble proteins was detected by western blot using anti-α5 (A11) and anti-β1 (TS2/16) in non-reducing conditions. (D) Luminescence of purified ectodomain of α5β1IAS with (clasped) or without (unclasped) the ACID-BASE coiled coil and in the absence (CTL) or presence of immobilized BSA or fibronectin (FN) under resting (Ca^2+^) or activating (Mn^2+^) metal ion conditions. Data are mean ± SD of three independent experiments. Statistical analysis: one-way ANOVA and Bonferroni’s post hoc analysis.

Endothelial β1 integrins are required for physiological angiogenesis both in the embryo^14^ and postnatally.^15^ Furthermore, the regulation of the conformational activation of endothelial β1 integrins is crucial for the formation of functional vascular networks^16^^,^^17^ and pathologically increased in abnormal cancer blood vessels.^18^ The identification of signaling pathways controlling β1 integrin conformational activation in ECs may support the design of vascular normalization strategies for cancer therapy.^19^^,^^20^ Hence, we sought to rationally design and generate a biochemical sensor capable of perceiving the conformational activation of endothelial β1 integrins and then exploit it in siRNA HTSs. We identified a functionally critical positional change within the β1 integrin extracellular domain that, without altering the receptor adhesive capacity, may be coupled to and revealed by a reversible and effective enzymatic system. In this regard, the key conformational transition connecting the headpiece opening and the separation of the α and β subunits happening during integrin activation strictly depends on the about 60° oscillation through which the hybrid domain moves away and aligns with the βI domain.^12^^,^^13^ Thus, we chose to insert a reversibly activatable enzyme tag into the externally exposed S_79_-K_85_ loop (Figure 1A) that shifts coordinately with the outward swinging hybrid domain during β1 integrin activation.^21^ Short Myc and hemagglutinin (HA) tags have already been successfully inserted into the long serine-rich stretch that is present in this loop in *Drosophila* βPS integrins only.^22^^,^^23^ Moreover, 27–33 kDa tags were already inserted in the S_79_-K_85_ loop^24^ or in its vicinity^25^ without affecting the functional properties of β1 integrins. We selected the Promega NanoLuc Binary Technology (NanoBiT) split luciferase formed by a large (LgBiT) and a small (SmBiT) subunit as an enzymatic tag, which has an extremely low self-association affinity but is able to reconstitute the catalytically active NanoBiT luciferase enzyme and brightly luminesce when brought into close proximity^26^ (Figure 1B). We designed the luminescent β1 integrin activation sensor (β1IAS) by inserting the LgBiT and SmBiT subunits of the NanoBiT, separated by a 15-amino-acid-long Gly-Ser linker, into the S_79_-K_85_ loop of human β1 integrins (Figures 1A and 1B), aiming to reconstitute the NanoBiT luciferase enzymatic activity during the hybrid domain swing-out movement (Figure 1B). We first created a soluble C-terminally clasped extracellular portion of α5β1IAS, which promotes secretion and the inactive bent/closed conformation,^27^^,^^28^ detected by both α5 and β1 integrin antibodies (Figure 1C). To this end, we added to the C-terminal end of the α5 and β1IAS subunit an amino acid linker containing a tobacco etch virus (TEV) protease site and an acidic or basic peptide, respectively, which, by heterodimerizing and forming a disulfide bridge, give rise to a clasped coiled-coil structure^27^^,^^28^ (Figure 1C). On the other hand, digestion with TEV protease allowed the generation of a soluble unclasped extracellular portion of α5β1IAS (Figure 1C), known to favor the active extended/open conformation.^27^^,^^28^ Then, we measured the extent of luminescence emitted by equal amounts of the clasped or unclasped extracellular portion of α5β1IAS exposed to immobilized bovine serum albumin (BSA) or fibronectin (FN) in the presence of physiological (1 mM Ca^2+^ or 1 mM Mg^2+^) or activating (2 mM Mn^2+^) concentrations of metal ions (Figure 1D). We observed that under control conditions and in the presence of BSA, the unclasped extracellular portion of α5β1IAS emitted a higher amount of luminescence than its clasped counterpart (Figure 1D). In addition, the contact with the FN ligand stimulated a marked increase in the luminescent signal emitted by the extracellular portion of both clasped and unclasped α5β1IASs in the presence of physiological or activating concentrations of metal ions (Figure 1D). Thus, the recombinant extracellular portion of β1IAS behaves as an effective and reliable reporter of the β1 integrin conformational activation state.

**Figure 2:**
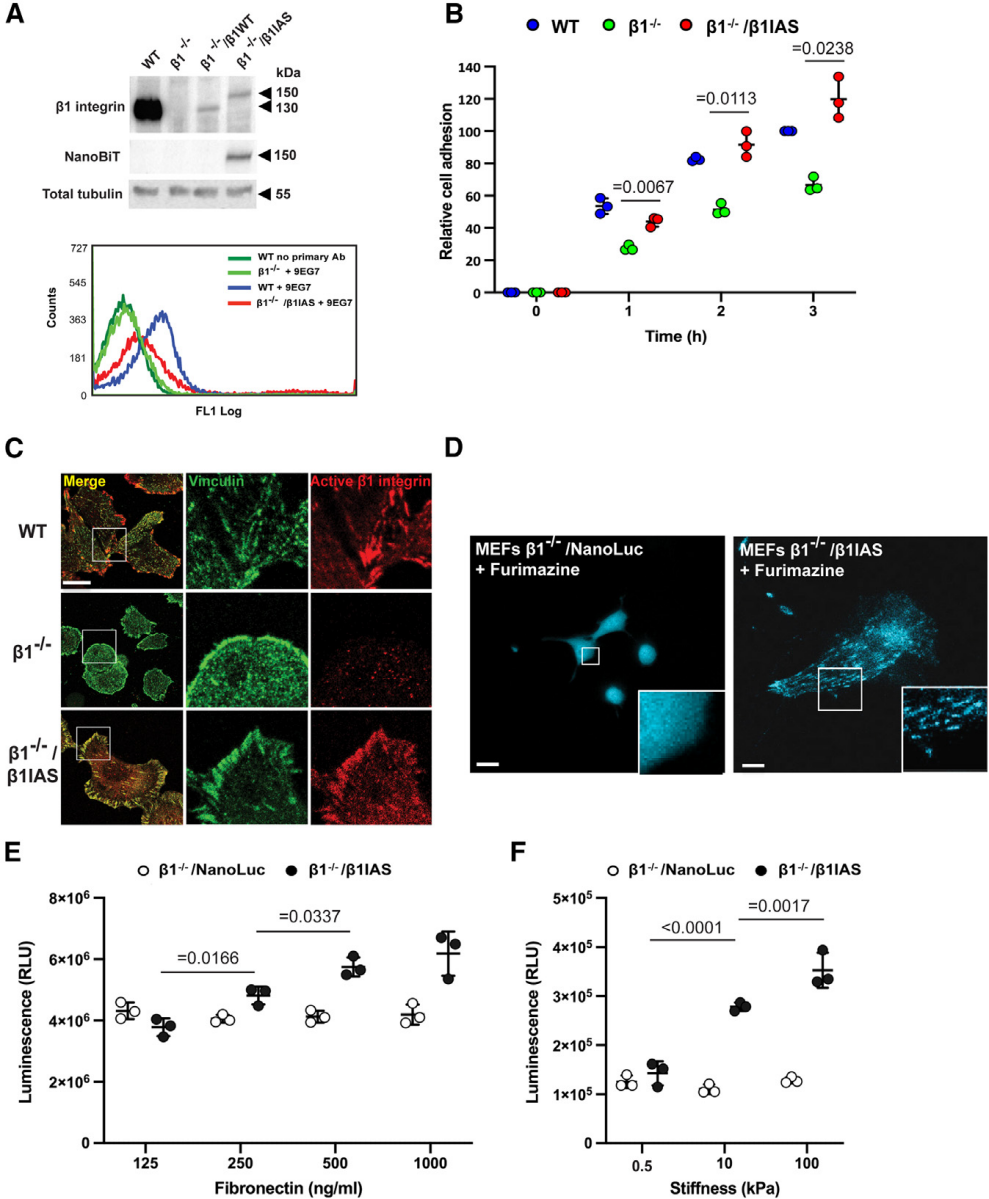
β1IAS functional characterization in *β1*^−/−^ MEFs (A) Top: western blot showing the expression of β1 integrin and NanoBiT in β1 integrin-null (*β1*^−/−^) mouse embryonic fibroblasts (MEFs) transduced with retroviral vector pLZRS-human WT β1 integrin (β1WT) or -β1IAS. Bottom: fluorescence-activated cell sorting (FACS) analysis using 9EG7 antibody in WT MEFs, *β1*^−/−^, and *β1*^−/−^ transduced with β1IAS (*β1*^−/−^/β1IAS). (B) Relative adhesion measured by the xCELLigence system in WT, *β1*^−/−^, and *β1*^−/−^/β1IAS MEFs plated on FN. Data are the mean ± SD of three independent experiments. Statistical analysis: two-way ANOVA and Bonferroni’s post hoc analysis. (C) Confocal microscopy showing vinculin (green) and 9EG7^+^ active β1 integrins (red) in WT, *β1*^−/−^, and *β1*^−/−^/β1IAS MEFs plated on FN. The left image insets highlight focal adhesion sites. Scale bar: 20 μm. (D) Luminescent microscopy in the presence of furimazine of *β1*^−/−^ MEFs transfected with either intact NanoLuc (left) or β1IAS (right) and plated on FN. Image insets show how most photons generated by β1IAS localize into *bona fide* focal adhesion sites (right) compared to the random localization of intact NanoLuc (left). Low-magnification (left) scale bars: 20 μm; high-magnification (right) scale bars: 5 μm. (E) Luminescence of *β1*^−/−^/β1IAS MEFs adhering for 30 min to increasing amounts (125–1,000 ng/mL) of FN compared to *β1*^−/−^ MEFs transfected with intact NanoLuc (*β1*^−/−^/NanoLuc). Data are mean ± SD of three independent experiments. Statistical analysis: two-way ANOVA and Bonferroni’s post hoc analysis. (F) Luminescence of *β1*^−/−^/β1IAS MEFs adhering for 30 min to FN-coated, increasingly stiff (0.5, 10, and 100 kPa) PAGEs compared to *β1*^−/−^ MEFs transfected with intact NanoLuc (*β1*^−/−^/NanoLuc). Data are mean ± SD of three independent experiments. Statistical analysis: two-way ANOVA and Bonferroni’s post hoc analysis.

Then, we retrovirally transduced the cDNA construct encoding for human β1IAS into β1 integrin-knockout mouse embryonic fibroblasts^29^ (*β1*^−/−^/β1IAS MEFs), comparing it with human wild-type β1 integrin (*β1*^−/−^/β1WT MEFs), and verified its surface exposure by fluorescence flow cytometry (Figure 2A). Moreover, as assessed by electric impedance sensing, β1IAS transduction effectively rescued the capability of *β1*^−/−^ MEFs to adhere to the main α5β1 integrin ligand FN (Figure 2B). In addition, confocal microscopy showed that transduced β1IAS physiologically localizes in vinculin containing ECM adhesions of *β1*^−/−^/β1IAS MEFs (Figure 2C). Notably, with luminescent microscopy, we detected photon signals specifically emanating from *bona fide* ECM adhesions of *β1*^−/−^/β1IAS MEFs, but not *β1*^−/−^ MEFs, retrovirally transduced with intact NanoLuc (*β1*^−/−^/NanoLuc MEFs) plated on FN (Figure 2D). To directly evaluate the ability of β1IAS to report the conformational activation of β1 integrins as a luminescence sensor, we challenged *β1*^−/−^/β1IAS MEFs with activating stimuli and employed *β1*^−/−^/NanoLuc MEFs as a negative control. We observed that plating the same number of cells on increasing amounts of FN for 1 h resulted in a proportional rise in the luminescence of *β1*^−/−^/β1IAS MEFs but not control *β1*^−/−^/NanoLuc MEFs (Figure 2E). Analogously, we detected an augmenting luminescent signal when *β1*^−/−^/β1IAS MEFs, but not *β1*^−/−^/NanoLuc MEFs, were grown on increasing stiffness hydrogels (Figure 2F). Therefore, functionally behaving as a WT β1 integrin, β1IAS represents an unprecedented luminescent sensor that allows noninvasive and quantitative measurements of β1 integrin conformational activation in living cells.

**Figure 3:**
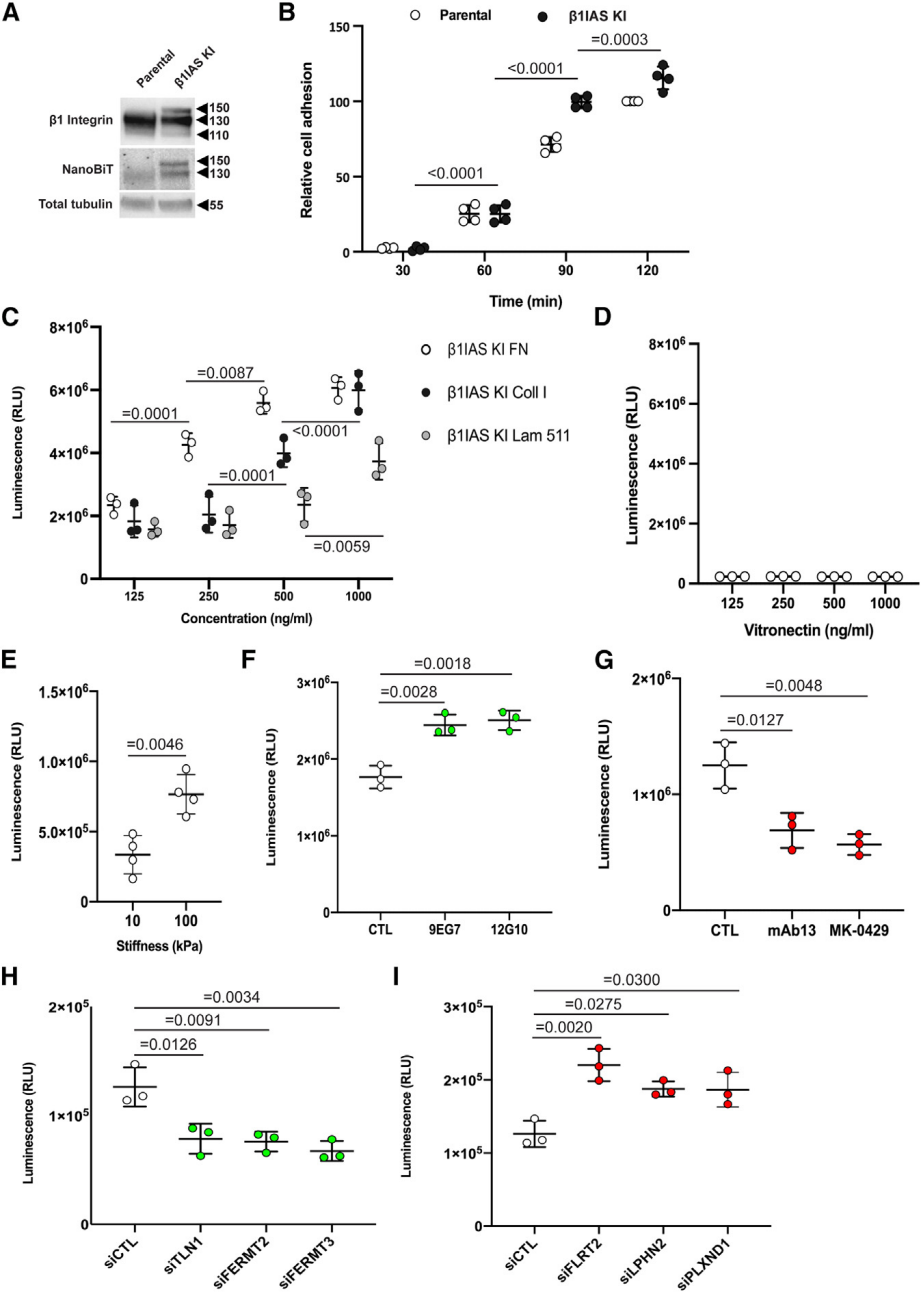
β1IAS functional characterization in genetic β1IAS KI TeloHAECs (A) Western blot showing the expression of β1 integrin and NanoBiT tag in β1IAS knockin (KI) ECs compared to WT TeloHAECs (parental). (B) Relative adhesion measured by the xCELLigence system in β1IAS KI ECs plated on FN compared to parental ECs. Data are the mean ± SD of four independent experiments. Statistical analysis: two-way ANOVA and Bonferroni’s post hoc analysis. (C) Luminescence of β1IAS KI ECs adhering for 30 min to increasing amounts (125–1,000 ng/mL) of FN, collagen type I (Coll I), or laminin 511 (Lam 511). Data are mean ± SD of three independent experiments. Statistical analysis: two-way ANOVA and Bonferroni’s post hoc analysis. (D) Luminescence of β1IAS KI ECs adhering for 30 min to increasing amounts (125–1,000 ng/mL) of vitronectin (VN). Data are mean ± SD of three independent experiments. Statistical analysis: one-way ANOVA and Bonferroni’s post hoc analysis. (E) Luminescence of β1IAS KI ECs adhering for 30 min to FN-coated, increasingly stiff (10 and 100 kPa) PAGEs. Data are mean ± SD of four independent experiments. Statistical analysis: two-tailed heteroscedastic Student’s t-test. (F) Luminescence of β1IAS KI ECs adhering for 15 min to 500 ng/mL FN in the presence of 9EG7 and 12G10 antibodies. Data are mean ± SD of three independent experiments. Statistical analysis: one-way ANOVA and Bonferroni’s post hoc analysis. (G) Luminescence of β1IAS KI ECs adhering for 15 min to 500 ng/mL FN in the presence of mAb13 or the pan-αv integrin and α5β1 antagonist MK-0429 (100 μM). Data are mean ± SD of three independent experiments. Statistical analysis: one-way ANOVA and Bonferroni’s post hoc analysis. (H) Luminescence of control β1IAS KI ECs (siCTL) and β1IAS KI ECs silenced for TLN1, FERMT2, and FERMT3 and then plated on 500 ng/mL FN. Data are mean ± SD of three independent experiments. Statistical analysis: one-way ANOVA and Bonferroni’s post hoc analysis. (I) Luminescence of control β1IAS KI ECs (siCTL) and β1IAS KI ECs silenced for FLRT2, LPHN2, and PLXND1 and then plated on 500 ng/mL FN. Data are mean ± SD of three independent experiments. Statistical analysis: one-way ANOVA and Bonferroni’s post hoc analysis.

Next, to chart signaling pathways responsible for the dynamic tuning of β1 integrin conformational activation in living human ECs, we generated heterozygous knockin (KI) immortalized primary ECs (TeloHAECs) stably expressing β1IAS (β1IAS KI ECs). Using the CRISPR-Cas9 system, we inserted a construct encoding for NanoBiT LgBiT and SmBiT subunits, separated by a 15-amino-acid-long Gly-Ser linker, into the S_79_-K_85_ loop (Figure 1A), encoded by exon 4, of the human β1 integrin (*ITGB1*) gene of parental WT TeloHAECs and isolated a β1IAS KI EC clone (Figure 3A). The NanoLuc antibody revealed that the NanoBiT/Gly-Ser linker insertion (≈20 kDa) was integrated in both the precursor (transforming it from 110 to 130 kDa) and mature (transforming it from 130 to 150 kDa) forms of β1 integrin. The three bands (of 110, 130, and 150 kDa) detected by the β1 integrin antibody in the β1IAS KI EC clone (Figure 3A) confirmed the heterozygosity of the clonal cell population. As its WT counterpart, the β1IAS subunit effectively dimerized with integrin α subunits, e.g., α2, α5, and αv (Figure S1). β1IAS KI ECs adhered to FN over time as WT TeloHAECs (Figure 3B). As observed with *β1*^−/−^/β1IAS MEFs (Figure 2E), we detected a proportionally growing luminescent signal when β1IAS KI ECs were allowed to adhere for 30 min to increasing concentrations of FN (Figure 3C, white). We also detected a proportionally growing luminescent signal when β1IAS KI ECs were allowed to adhere for 30 min on increasing concentrations of type I collagen (Coll I) (Figure 3C, black) and laminin 511 (Lam 511)^30^ (Figure 3C, gray), which are the main ligands of α2β1 and α6β1 integrins, respectively, thus supporting the exploitability of β1IAS in revealing the conformational activation of other β1 integrin dimers than the FN receptor α5β1 integrin in human ECs. We did not detect a luminescent signal when β1IAS KI ECs were allowed to adhere for 30 min to increasing concentrations of vitronectin (VN) (Figure 3D), the main ECM ligand of αvβ5 and αvβ3 integrins, thus supporting the specificity of β1IAS in revealing β1 integrin activation only in human ECs. To further characterize the specificity of β1IAS in detecting integrin-dependent adhesion, we plated β1IAS KI ECs for 30 min on poly-D-lysine (1 mg/mL) and did not detect a background luminescent signal, compared to FN (Figure S2). Like *β1*^−/−^/β1IAS MEFs (Figure 2F), when grown on hydrogels with increasing stiffness, β1IAS KI ECs also emitted proportionally rising luminescence (Figure 3E). To further functionally characterize β1IAS KI ECs, we took advantage of 9EG7^31^^,^^32^ and 12G10^32^^,^^33^ monoclonal antibodies (mAbs) that specifically recognize and stabilize the active conformation of β1 integrins. To maximize the capability of detecting a positive modulation of β1 integrin activation, the cells were allowed to adhere to the same amount of FN for 15 min, in the presence or absence of the two mAbs. Of note, we documented how β1 integrin-activating mAbs significantly increase the luminescent signal emitted by β1IAS KI ECs adhering to FN (Figure 3F). In contrast, to evaluate the exploitability β1IAS to detect β1 integrins in their inactive state, we used mAb13, which specifically recognizes and stabilizes the inactive conformation,^32^^,^^34^ and the inhibitor MK-0429, which behaves as a potent pan-αv integrin and α5β1 integrin antagonist.^35^^,^^36^^,^^37^ We let β1IAS KI ECs adhere to the same amount of FN for 15 min, in the presence or absence of mAb13 or MK-0429. Both inhibiting stimuli significantly decreased the extent of luminescent signal emitted by β1IAS KI ECs adhering to FN (Figure 3G). Furthermore, we observed that siRNA-mediated silencing of well-known activators of integrin function, such as talin-1 (TLN1), kindlin-2 (FERMT2), and kindlin-3 (FERMT3) adaptor proteins^9^^,^^10^ (Figure S3A), results in a significant drop of the luminescent signal of β1IAS KI ECs allowed to adhere to FN for 1 h, compared to β1IAS KI ECs silenced with control siRNA (Figure 3H), without affecting cell viability (Figure S3B). The silencing of FN leucine-rich transmembrane protein 2 (FLRT2) ligand^38^ or its receptor latrophilin 2 (LPHN2)^38^ or the semaphorin receptor plexin D1 (PLXND1),^39^ which we^19^^,^^38^ and others^39^^,^^40^ found to inhibit, via different mechanisms, the function of the major integrin activating small GTPase Rap1,^41^^,^^42^ caused instead a robust elevation of β1IAS KI EC luminescence (Figure 3I), without affecting cell viability (Figure S3B). Those data substantiate the successful generation of an EC clone constitutively expressing an effective sensor of the conformational activation state of β1 integrin and its potential exploitability in HTSs to identify signaling pathways or drugs capable of positively or negatively modulating β1 integrin activation and function in living ECs.

### Identification of key molecular pathways controlling β1 integrin activation in ECs

**Figure 4:**
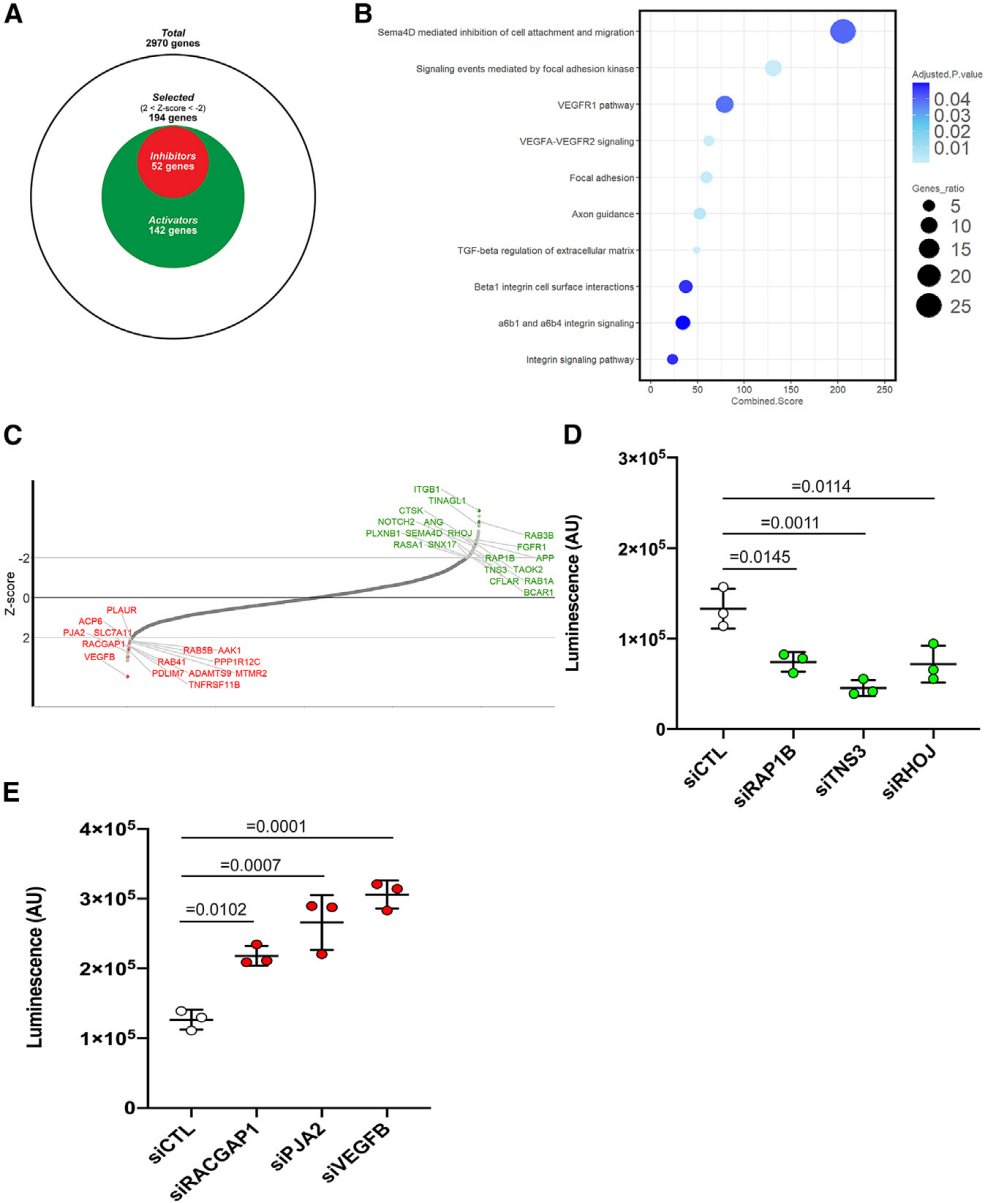
siRNA HTS in β1IAS TeloHAECs to identify activators and inhibitors of β1 integrin (A) Schematic of the high-throughput screening (HTS)-selected genes (194), among the 2,970 genes expressed by parental WT TeloHAECs, whose silencing in β1IAS KI ECs induces a decreased luminescent signal (*Z* score < −2), therefore β1 integrin activators (142, in green), and those whose silencing induces an increased luminescent signal (*Z* score > 2), therefore β1 integrin inhibitors (52, in red). (B) Bubble plot representing top integrin focused enriched pathways (adjusted *p* < 0.05) based on candidate genes obtained from the HTS (2 < *Z* score < −2). All enriched pathways are listed in Table S2. The EnrichR combined score is the log of the *p* value from the Fisher exact test multiplied by the *Z* score of the deviation from the expected rank. Bubble color (adjusted *p* value) was computed using the Benjamini-Hochberg method for correction for multiple hypotheses testing. The gene ratio is the overlap between the input list and the gene sets in each gene set library for ranking a pathway’s relevance to the input list. (C) Luminescent intensity *Z* score mean of three biological replicates for each endothelial gene whose siRNA was contained in the Qiagen Druggable Genome v.3 siRNA library. The listed genes (β1 integrin inhibitors in red and β1 integrin activators in green) were chosen for secondary validation. (D) Luminescence of control β1IAS KI ECs (siCTL) and β1IAS KI ECs silenced for RAP1B, TNS3, and RHOJ and then plated on 500 ng/mL FN. Data are mean ± SD of three independent experiments. Statistical analysis: one-way ANOVA and Bonferroni’s post hoc analysis. (E) Luminescence of control β1IAS KI ECs (siCTL) and β1IAS KI ECs silenced for RACGAP1, PJA2, and VEGF-B and then plated on 500 ng/mL FN. Data are mean ± SD of three independent experiments. Statistical analysis: one-way ANOVA and Bonferroni’s post hoc analysis.

To assess whether and how the scaling and proportional emission of luminescence by β1IAS KI ECs may be exploited to pinpoint previously uncharacterized regulators of endothelial β1 integrin conformational activation, we conducted a luminescent siRNA HTS using a human Druggable Genome v.3 siRNA library. RNA sequencing (RNA-seq) analysis filtered 3,978 out of 6,948 druggable genome library siRNAs, whose mRNAs were not actively transcribed in β1IAS KI ECs. In the HTS, 2,970 expressed genes were silenced in β1IAS KI ECs, and the resulting luminescence emission data were measured and analyzed, unveiling 194 statistically significantly candidate genes that emerged as positive (142 genes, *Z* score < −2) or negative (52 genes, *Z* score >2) regulators of β1 integrin activation (Figure 4A). Bioinformatic analysis of candidate genes, performed through an EnrichR web-based tool used to combine KEGG, WikiPathway, NCI-Nature, and BioPlanet databases, identified as top enriched pathways those governing integrin-based focal adhesion formation and signaling, along with vascular endothelial growth factor (VEGF) signaling, axon guidance, and integrin cell surface interactions and signaling (Figures 4B and S4; Table S2). In addition to ITGB1 itself, among β1IAS positive regulators, we identified genes encoding for proteins known to promote (1) β1 integrin conformational activation, e.g., the talin activating small GTPase RAP1B,^42^ the β1 integrin adaptor tensin 3 (TNS3),^43^ the reported β2 integrin activator cathepsin K (CTSK),^44^ and the α5β1 integrin ligand tubulointerstitial nephritis antigen-like 1 (TINAGL1)^45^; (2) β1 integrin endosomal recycling, e.g., sorting nexin 17 (SNX17^29^^,^^46^), RAS p21 protein activator 1 (RASA1, also known as [aka] p120RASGAP),^47^^,^^48^ and RAB1A^49^; (3) focal adhesion formation, e.g., RHOJ,^50^ BCAR1 scaffold protein, Cas family member (BCAR1, aka p130Cas),^51^ and the BCAR1/p130Cas interactor CASP8 and FADD-like apoptosis regulator (CFLAR)^52^; and (4) angiogenesis, e.g., fibroblast growth factor receptor 1 (FGFR1),^53^^,^^54^ angiogenin (ANG),^55^ and semaphorin 4D (SEMA4D)^56^^,^^57^ and its receptor PLXNB1,^56^^,^^57^ which we previously showed to promote blood vessel formation by recruiting the MET tyrosine kinase receptor^57^ (Figure 4C). On the other hand, beyond the few already described negative regulators of β1 integrin activation and function, such as urokinase plasminogen activator receptor (PLAUR, aka uPAR)^58^ and Rac GTPase-activating protein 1 (RACGAP1),^59^ we pinpointed several hitherto unknown β1 integrin inhibitors (Figure 4C), among which Praja ring finger ubiquitin ligase 2 (PJA2) and VEGFB. Silencing some (Figure S3A) of these candidate activators (RAP1B, TNS3, and RHOJ) and inhibitors (RACGAP1, PJA2, and VEGFB) identified in our HTS consistently resulted in a clear reduction (Figure 4D) or increase (Figure 4E) in the luminescent signal of β1IAS KI ECs, thus confirming the robustness of the experimental approach. Silencing those proteins in ECs does not affect cell viability (Figure S3B) except VEGFB silencing, being VEGF-B a survival factor.^60^

### The E3 ubiquitin ligase Pja2 promotes kindlin-2 degradation and inhibits β1 integrin activation in ECs

**Figure 5:**
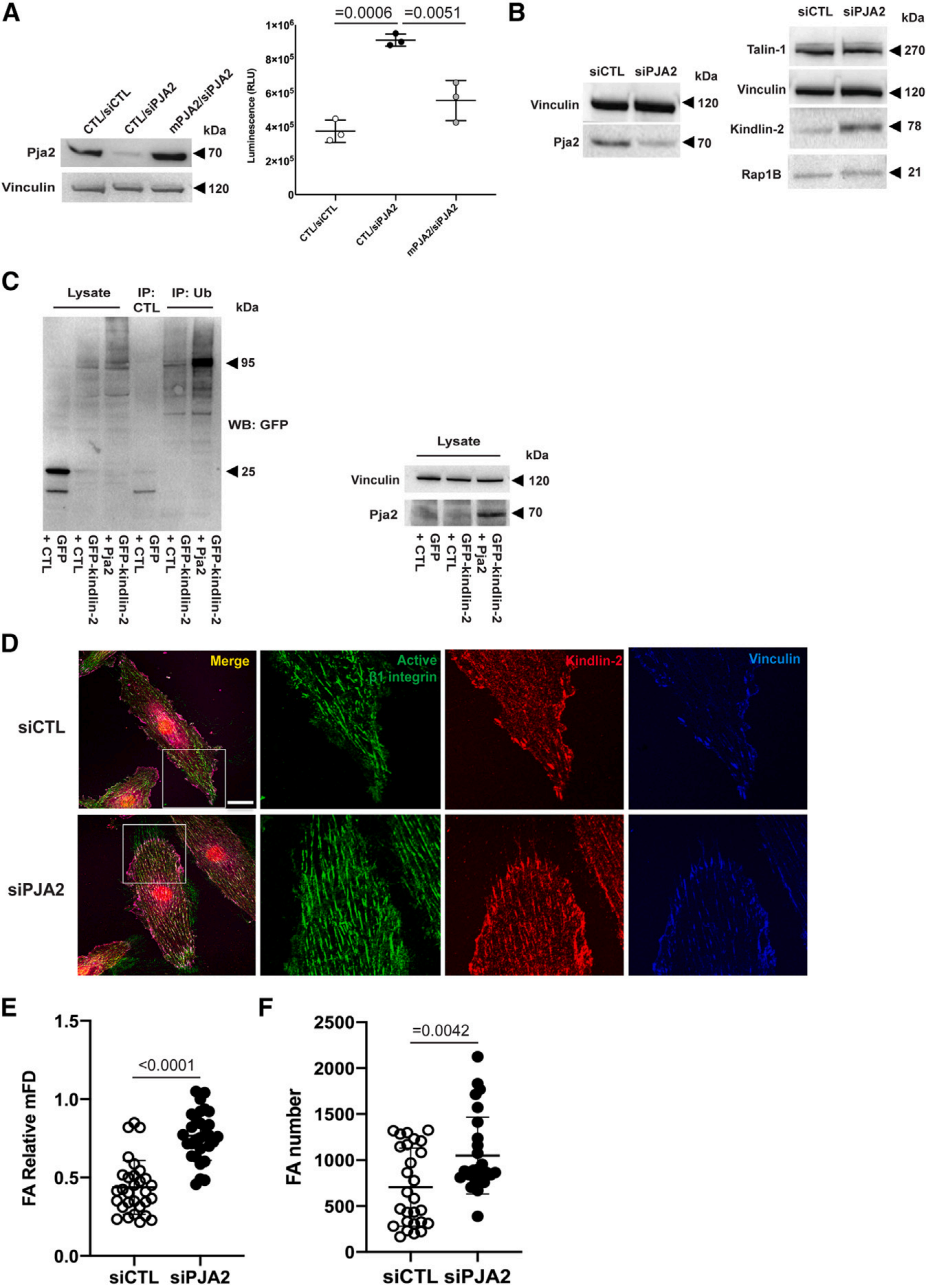
The E3 ubiquitin ligase PJA2 promotes kindlin-2 degradation and inhibits β1 integrin activation in ECs (A) Left: western blot showing the expression of PJA2 in WT TeloHAECs after PJA2 silencing and silenced cells transduced with silencing resistant murine PJA2 (mPJA2). Right: luminescence intensity of β1IAS KI ECs plated on 500 ng/mL FN, silenced for PJA2, and rescued with mPJA2. Data are mean ± SD of three independent experiments. Statistical analysis: one-way ANOVA and Bonferroni’s post hoc analysis. (B) Western blot showing the expression of kindlin-2, talin-1, and Rap1B in WT TeloHAECs after PJA2 silencing. (C) Left: western blot showing ubiquitinated GFP kindlin-2 pulled down by ubiquitin affinity beads in Phoenix cells overexpressing PJA2 or control construct. The first lane corresponds to the incubation of lysate from cells overexpressing control constructs on non-ubiquitinated beads (see STAR Methods). Right: western blot showing the expression of PJA2 in cells used in the ubiquitinated assay shown on the left. (D) Confocal microscopy showing 9EG7^+^ active β1 integrin (green), kindlin-2 (red) and vinculin (blue) in WT TeloHAECs plated on 1.5 μg/mL FN and silenced for PJA2. The image insets highlight focal adhesion sites. Scale bar: 20 μm. (E) Relative maximum Feret diameter (mFD) of adhesion sites (FA) in WT TeloHAECs silenced for PJA2 compared to siCTL. Data are the mean ± SD of three independent experiments (10 cells each). Statistical analysis: two-tailed heteroscedastic Student’s t test. (F) Number of adhesion sites (FA) in siCTL and siPJA2 ECs as in (E). Data are the mean ± SD of three independent experiments (9 cells each). Statistical analysis: two-tailed heteroscedastic Student’s t test.

The rise in luminescence caused by the loss of PJA2 in human β1IAS KI ECs was efficiently diminished by transduction with silencing resistant murine Pja2 (mPJA2) (Figure 5A). Pja2 is an E3 ubiquitin ligase originally identified for its ability to promote the degradation of the regulatory subunit of protein kinase A (PKA),^61^ which can behave as a force-dependent binding partner of talin-1 via its regulatory subunit.^62^ Additionally, Pja2 fosters the ubiquitin-dependent proteolysis of monopolar spindle-one-binder protein 1 (Mob1), which inhibits the transcriptional regulator yes-associated protein 1 (Yap1).^63^^,^^64^ Since in this context, kindlin-2 acts as an adaptor protein, promoting the interaction between Pja2 and Mob1,^63^ we wondered whether Pja2 may inhibit β1 integrin activation (Figures 4C and 4E) by, at least in part, downregulating kindlin-2 levels. Indeed, western blot analysis revealed that the silencing of PJA2 in WT TeloHAECs increases the amounts of kindlin-2, leaving the levels of both talin-1 and Rap1B unchanged (Figure 5B). Importantly, Pja2 overexpression significantly augmented the polyubiquitination of kindlin-2 (Figure 5C), thus suggesting that Pja2 may negatively regulate β1 integrin activation by fostering the ubiquitin-dependent proteolysis of kindlin-2. Moreover, fluorescence confocal microscopy analysis of adhesion sites containing kindlin-2, vinculin, and conformationally active β1 integrin, recognized by the 9EG7 mAb (Figure 5D), showed that the lack of Pja2 increases the adhesion site Feret diameter (Figure 5E) and number (Figure 5F) in WT TeloHAECs. Those data suggest that Pja2 negatively regulates β1 integrin activation and focal adhesion formation by decreasing kindlin-2 protein levels.

### VEGF-B is an effective inhibitor of β1 integrin activation in ECs

**Figure 6:**
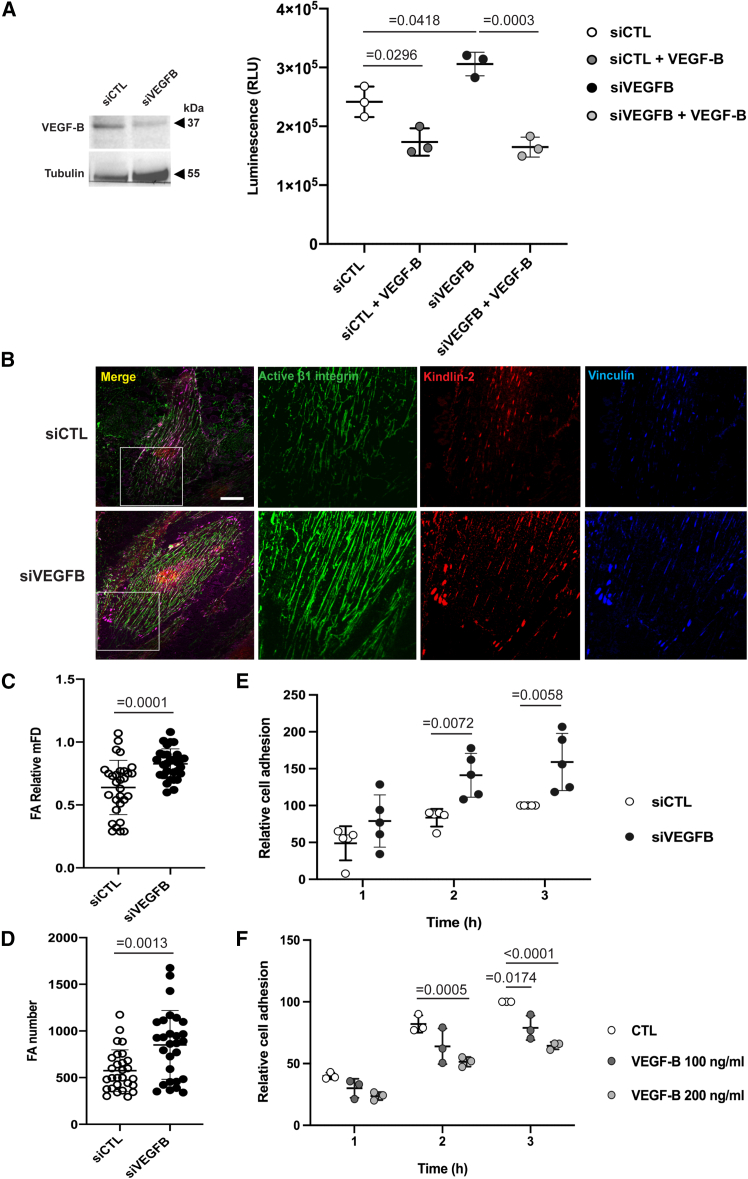
VEGF-B is an effective inhibitor of β1 integrin activation in ECs (A) Left: western blot showing the expression of VEGF-B protein in WT TeloHAECs after VEGFB silencing compared to siCTL. Right: luminescence of TeloHAEC β1IAS plated on 500 ng/mL FN and silenced for VEGFB in the presence or absence of exogenous VEGF-B for 15 min. Data are mean ± SD of three independent experiments. Statistical analysis: one-way ANOVA and Bonferroni’s post hoc analysis. (B) Confocal microscopy showing 9EG7^+^ active β1 integrin (green), kindlin-2 (red), and vinculin (blue) in WT TeloHAECs plated on 1.5 μg/mL FN and silenced for VEGFB. The image insets highlight focal adhesion sites. Scale bar: 20 μm. (C) Relative maximum Feret diameter (mFD) of adhesion sites (FA) in WT TeloHAECs silenced for VEGFB compared to siCTL. Data are the mean ± SD of three independent experiments (10 cells each). Statistical analysis: two-tailed heteroscedastic Student’s t test. (D) Number of adhesion sites (FA) in WT TeloHAECs silenced for VEGFB compared to siCTL. Data are the mean ± SD of three independent experiments (9 cells each). Statistical analysis: two-tailed heteroscedastic Student’s t test. (E) Relative adhesion measured by the xCELLigence system in WT TeloHAECs plated on 1.5 μg/mL FN and silenced for VEGFB. Data are the mean ± SD of five independent experiments. Statistical analysis: two-way ANOVA and Bonferroni’s post hoc analysis. (F) Relative adhesion measured by the xCELLigence system in WT TeloHAECs plated on 1.5 μg/mL FN and treated or not with 100 and 200 ng/mL exogenous VEGF-B. Data are the mean ± SD of three independent experiments. Statistical analysis: two-way ANOVA and Bonferroni’s post hoc analysis.

**Figure 7:**
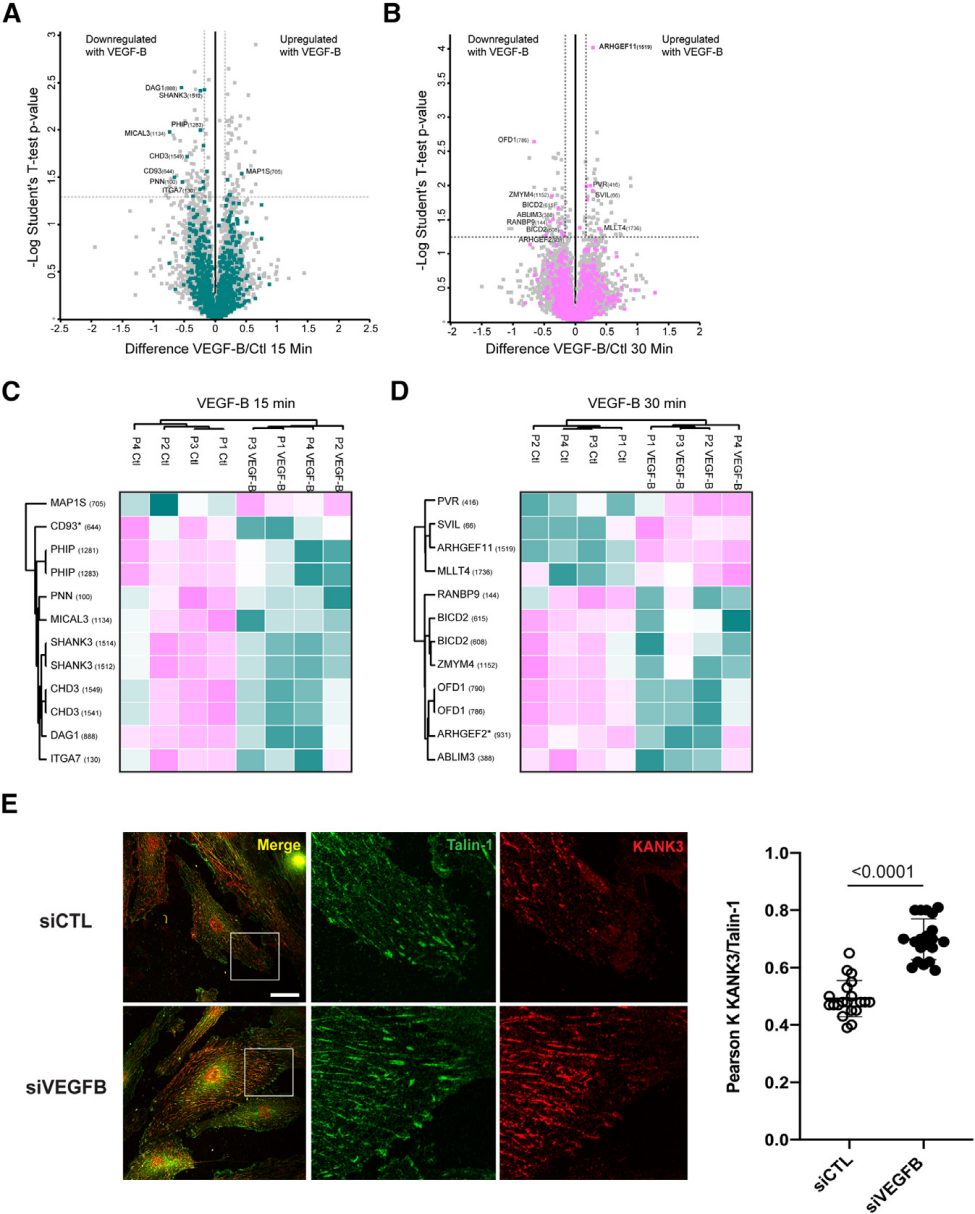
VEGF-B modulates the phosphorylation of CMSC mediators (A and B) Volcano plots of the phosphoproteome of parental WT TeloHAECs stimulated with VEGF-B or control for 15 (A) or 30 (B) min. *n* = 4 biological replicates. Colored dots are phosphorylation sites of proteins annotated to the GOBP categories “cell adhesion” or “cytoskeleton organization.” Dashed bars separate significantly regulated sites with *p* ≤ 0.05 and difference ≥ ±0.2. The position of the phosphorylation site within the protein sequence is in parentheses following the gene name. (C and D) Heatmaps of the up- and downregulated sites highlighted in the volcano plots in (A) and (B), respectively. Colors are based on the intensity values measured for the phosphorylated peptide by MaxQuant; purple represents upregulation upon VEGF-B stimulation, and green represents downregulation upon VEGF-B stimulation. An ^∗^ indicates a known regulatory site. (E) Left: confocal microscopy showing talin-1 (green) and KANK3 (red) in WT TeloHAECs plated on 1.5 μg/mL FN and silenced for VEGFB. The image insets highlight contact sites between talin-1^+^ adhesions and the CMSC mediator KANK3. Scale bar: 20 μm. Right: Pearson correlation between talin-1^+^ adhesions and the CMSC mediator KANK3. Data are the mean ± SD of two independent experiments (10 cells each). Statistical analysis: two-tailed heteroscedastic Student’s t test.

ECs express high levels of autocrine VEGF-B that signals, at least in part, via VEGFR-1, a tyrosine kinase receptor uniquely endowed with extremely weak enzymatic activity.^60^ Our unbiased HTS identified VEGFB as the strongest inhibitor of β1 integrin activation, among genes silenced in β1IAS KI ECs (Figures 4C and 4E), in agreement with VEGF-B's reported role as an anti-angiogenic^65^^,^^66^ and anti-tumor^66^^,^^67^ factor. Consistently, stimulation with exogenous VEGF-B rescued the increased luminescence stimulated by endogenous VEGFB silencing in β1IAS KI ECs (Figure 6A). In addition, confocal fluorescence microscopy revealed how, compared with WT TeloHAECs, siVEGFB cells display an increased Feret diameter and number of adhesion sites in which kindlin-2, vinculin, and 9EG7^+^ active β1 integrins localize (Figures 6B–6D). Impedance analysis also demonstrated that the lack of endogenous VEGF-B in WT TeloHAECs is accompanied by increased cell adhesion and spreading on FN (Figure 6E) and, conversely, how incubation with increasing amounts of exogenous VEGF-B decreases WT TeloHAEC adhesion to and spreading on FN in a concentration-dependent manner (Figure 6F). As the biochemical and molecular mechanisms that VEGF-B employs to signal through VEGFR-1 and/or other membrane receptors are still incompletely defined,^60^ we analyzed by mass spectrometry the phosphoproteome changes in WT TeloHAECs stimulated with exogenous VEGF-B for 15 and 30 min. We found that exogenous VEGF-B induced changes in the phosphorylation levels of hundreds of sites (Figures 7A and 7B). Because of the effects of VEGF-B on cell adhesion, we focused on changes in proteins involved in cell adhesion and cytoskeleton organization (based on Gene Ontology annotation). After 15 min stimulation, VEGF-B negatively regulated the phosphorylation of the majority of the sites belonging to proteins involved in those functions (Figures 7A and 7C). The most downregulated sites included SHANK3, which, by competing with talin for Rap1-GTP binding, impairs Rap1-GTP/talin-driven integrin activation,^38^^,^^68^ and MICAL3, a key component of the ECM adhesion-associated cortical microtubule stabilization complexes (CMSCs)^69^^,^^70^ that, via KANK proteins, interact with and activate talin and associated integrins^71^ (Figure 7A). Similar results were obtained after 30 min stimulation with VEGF-B (Figures 7B and 7D). Consistently, confocal fluorescence microscopy analysis showed that the lack of endogenous VEGF-B results in a sizable increase of KANK3 colocalization with talin-1 and 9EG7^+^ active β1 integrins at adhesion sites (Figure 7E) of WT TeloHAECs.

## Discussion

Allosteric transitions between inactive and active conformations allow integrins to fulfill their key roles in multiple physiological and pathological contexts.^2^^,^^3^^,^^4^^,^^5^^,^^6^^,^^7^^,^^8^ Structural studies^12^^,^^13^^,^^21^^,^^27^^,^^28^^,^^72^ and the exploitation of mAbs recognizing conformation-specific epitopes^31^^,^^32^^,^^34^^,^^73^^,^^74^ defined the molecular details of the dynamic functional behavior of integrins. However, extracellular secreted factors^6^^,^^54^^,^^75^^,^^76^ and downstream signal transduction pathways^19^^,^^39^^,^^40^ that modulate integrin allosteric equilibrium^9^^,^^10^^,^^77^ have been only in part characterized. Thus, developing bioorthogonal approaches to monitor integrin conformational activation and inhibition in living cells and organisms may allow a wider and more comprehensive deciphering of the biochemical and cellular mechanisms that regulate integrin function *in vivo*, with the potential for extension into therapeutics.^19^

We report the rational design and implementation of β1IAS, a luminescent sensor that allows effective and noninvasive monitoring of the conformational activation state of β1 integrins in living cells. Such a sensor was conceived to detect the oscillatory outward movement of the β1 integrin hybrid domain, which is ultimately responsible for the functional connection between the headpiece opening and the separation of the α and β subunits of the heterodimer,^12^^,^^13^ which, respectively, enable integrins to interact with ECM ligands, e.g., FN, and cytoskeletal adaptors, such as talin and kindlin.^9^^,^^10^ Since the swing out of the β subunit hybrid domain is shared by allosteric dynamics driving the conformational activation and/or the binding to ligands of all the integrins described so far,^13^ it is conceivable that the molecular strategy underpinning β1IAS may be harnessed to generate as many integrin activation sensors. In this regard, the extremely low mutual affinity of LgBiT and SmBiT^26^ incorporated within the S_79_-K_85_ loop, which moves coordinately with the outward swinging hybrid domain,^21^ together with its reversibility, appears to let β1IAS report the allosteric activation of β1 integrin noninvasively and accurately.

Our analyses on the recombinant extracellular domain of α5β1IAS and β1IAS either transduced in *β1*^−/−^ MEFs or genetically expressed in β1IAS KI ECs illustrate how β1IAS acts as a sensor that perceives the modulation of the conformational activation state of β1 integrin by leg separation, ligand binding, metal ions, and ECM ligand specificity, amounts, and stiffness. Moreover, in luminescence microscopy, the β1IAS active conformer glows photons in discrete plasma membrane areas that are similar to *bona fide* ECM adhesion sites. β1IAS also proved a faithful reporter of β1 integrin allosteric modulation upon treating cells either with mAbs known to recognize and stabilize active (9EG7^31^^,^^32^ and 12G10^32^^,^^33^) or inactive (mAb13)^32^^,^^34^ β1 integrins or with the pan-αv integrin and α5β1 integrin antagonist inhibitor MK-0429^35^^,^^36^^,^^37^ or after silencing β1 integrin activators^9^^,^^10^^,^^22^^,^^41^ (TLN1, FERMT2, and FERMT3) or inhibitors^38^^,^^39^^,^^40^^,^^75^ (FLRT2, LPHN2, and PLXND1). In sum, β1IAS, both as a recombinant protein and in live cells, behaves as an effective luminescent reporter of the β1 integrin conformational activation state in response to multiple biochemical or physical cues.

Our unbiased interrogation in siRNA HTS of about 3,000 genes expressed in β1IAS KI ECs substantiated the validity and robustness of β1IAS but also proved how the sensor may be effectively exploited to identify regulators of the function and activation of β1 and, possibly, other integrins, with the perspective of scaling up the analysis. Indeed, bioinformatics of candidates emerged in the screening as genes coding for β1 integrin positive regulators identified those governing integrin-based focal adhesion formation and signaling, in addition to angiogenesis, as the most represented signaling pathways. Among the activators and positive regulators of β1 integrins, the siRNA HTS on β1IAS KI ECs fittingly identified ECM ligands (TINAGL1^45^), structural and signaling regulators and adaptors of integrin-based adhesion sites (TNS3,^43^ RHOJ,^50^ and BCAR1/p130Cas and its interactor CFLAR^52^), proteins involved in the recycling of endocytosed β1 integrin (RASA1/p120RASGAP^47^^,^^48^ and RAB1A^49^) and its rescue from lysosomal degradation (SNX17^29^^,^^46^), angiogenic factors and their signaling pathways (FGFR1,^53^^,^^54^ ANG,^55^ and SEMA4D and its receptor PLXNB1^56^^,^^57^), and the key small GTPase directly involved in the activation of talin-1 and integrins, namely RAP1B.^41^^,^^42^

The less grasped aspect of integrin allosteric regulation is the identification of inhibitory signaling pathways,^76^^,^^77^ whose modulation may also be exploited for therapeutic purposes.^11^^,^^19^ In addition to a few previously described negative regulators, e.g., PLAUR^58^ and RACGAP1,^59^ a number of hitherto undisclosed candidate β1 integrin inhibitors emerged from the siRNA HTS on β1IAS KI ECs. We identified PJA2 RING E3 ligase^61^ as an effective negative regulator of β1 integrin activation and function in ECs. Confirming and extending previous findings by Song and colleagues,^63^ we showed that Pja2 not only interacts with but also reduces the levels of the cytosolic and activating β1 integrin interactor kindlin-2 by promoting its ubiquitination-dependent degradation. We here discover kindlin-2 as a ubiquitinated target for Pja2, proposing its direct negative role on integrin activation modulation, together with its already known ubiquitinated target, the PKA regulatory subunit, which binds talin-1 in a force-dependent manner.^62^ It will be interesting to investigate whether the ubiquitinating activity of Pja2 on kindlin-2 might be promoted by mechanical stimuli.

Our screening pinpointed VEGFB as the most potent β1 integrin inhibitor among the endothelial genes silenced by the employed library. The inhibitory role that VEGF-B exerts on integrin-mediated EC adhesion to the ECM is consistent with what was reported in some studies on tumor angiogenesis^65^^,^^66^^,^^67^ but not on angiogenesis associated with the repair of damaged tissues, where VEGF-B would instead appear to promote angiogenesis.^60^ It has been proposed that, through VEGFR-1, VEGF-B may inhibit angiogenesis by counteracting FGFR-1 signaling,^66^ which our screening indeed identified as an activator of β1 integrin in ECs, consistent with previous reports.^53^^,^^54^ However, the signaling pathways regulating VEGFR-1 negative and FGFR-1 positive impacts on β1 integrin activation remain unclear. Our phosphoproteomic mass spectrometry identified the key CMSC component MICAL3^69^^,^^70^ among the substrates, whose phosphorylation is modulated by VEGF-B. CMSCs associate with ECM adhesion sites via the KANK3 adaptor, interacting with and activating talin and integrins.^69^^,^^70^^,^^71^ The observed increased colocalization among KANK3, talin, and active β1 integrin upon endogenous VEGFB silencing hints that the inhibitory function of VEGF-B may be mediated by the modulation of phosphorylation of CMSC components. Our data also suggest that VEGF-B may inhibit β1 integrin activation by regulating the phosphorylation of the talin inhibitor SHANK3.^38^^,^^68^

Among the candidate inhibitors of β1 integrin activation identified in our HTS study, we mention PDZ and LIM domain 7 (PDLIM7), protein phosphatase 1 regulatory subunit 12C (PPP1R12C), and myotubularin-related protein 2 (MTMR2) as examples of regulators of integrin conformation, mechanosensing, and traffic, respectively. Indeed, the lack of the cytoskeletal protein PDLIM7 in mice causes a prothrombotic phenotype and platelet dysfunction^78^ potentially due to the Rap1-dependent conformational hyperactivation of platelet αIIbβ3 integrin,^79^ as reported in PDLIM1-knockout mice.^80^ PPP1R12C is, instead, a ubiquitous myosin phosphatase target subunit that reduces actomyosin contractility^81^ and the formation and size of ECM adhesions.^82^ Finally, phosphatidylinositol 3 phosphate (PtdIns3P) phosphatase MTMR2, by dephosphorylating PtdIns3P,^83^ may counteract docking on early endosomes of SNX17 and the ensuing retrieval of endocytosed β1 integrin from lysosomal degradation.^29^^,^^46^

In conclusion, β1IAS represents a prototype of an effective noninvasive luminescent sensor that may be exploited in live cells to quantify the conformational activation of integrins because of their modulation by biochemical pathways, physical forces, traffic, and degradation, with possible therapeutic implications, as VEGF-B. The use of this sensor in broader screenings may allow a more detailed charting of the mechanisms governing the activation of β1 integrins, and potentially the other integrin β subunits, in different cultured cell types and tissue contexts of genetically modified animals. Even with the intrinsic limitations due to the long exposure time of luminescence microscopy, this type of sensor may enable a noninvasive study of the subcellular localization dynamics of active integrins. Finally, the strategy that underpins the creation of β1IAS could be exploited to monitor functionally relevant conformational changes of other receptors and transmembrane proteins in general, e.g., cadherins,^84^^,^^85^^,^^86^^,^^87^ plexins,^88^ LDL,^89^^,^^90^ and epidermal growth factor (EGF)^91^ receptors.

### Limitations of the study

Although we provided evidence for the sensitivity and exploitability of β1IAS by employing a number of diverse *in vitro* and *in vivo* biochemistry, cell biology, and microscopy assays, we cannot formally rule out the possibility that the insertion of NanoBiT split luciferase may affect, at least in part, the balance of β1 integrin conformational activation dynamics. Structural studies will be needed to define the details of how the two NanoBiT subunits interact during β1IAS transition between inactive and active conformations and investigate whether the presence of NanoBiT might affect β1IAS conformational activation dynamics when compared to the WT β1 integrin.

In addition, further investigation is needed to comprehensively characterize the mechanisms by which Pja2 and VEGF-B inhibit the conformational activation of β1 integrin in the vascular endothelium both in culture and *in vivo* models of, e.g., tumor angiogenesis. Moreover, HTS siRNA data showed that in ECs, β1IAS is more sensitive in detecting positive (142) than negative (52) regulators of β1 integrin conformational activation. This could reflect a real imbalance in the number of ratios between activators and inhibitors or be due to intrinsic biochemical features of β1IAS, whose very nature should be further investigated.

Finally, the HTS approach based on the use of a siRNA library, while allowing the identification of previously unknown and promising regulatory candidates of integrin β1 activation, is certainly not complete, being technically limited by both the number of silenced genes compared to those actively transcribed in ECs and the efficacy of gene silencing within the time frame of the protocol.

## Resource availability

### Lead contact

Further information should be directed to and will be fulfilled by the lead contact, Guido Serini (guido.serini@ircc.it).

### Materials availability

Plasmids and cell lines generated in this study can be shared upon request.

### Data and code availability

• Raw data, uncropped blots, and RNA-seq BAM files have been deposited at Figshare, and the mass spectrometry data are at the ProteomeXchange Consortium via the PRIDE^92^ partner repository and publicly available as of the date of publication. Accession numbers are listed in the key resources table.
• This paper does not report original code.
• Any additional information required to re-analyze the data reported in this paper is available from the lead contact upon request.

## Acknowledgments

This research has received funding from AIRC under IG 2018 - ID. 21315 - P.I. Serini Guido and IG 2023 - ID. 28763 - P.I. Serini Guido; FPRC-ONLUS grant “MIUR 2010 Vaschetto—5 per mille 2010 MIUR” (to G.S.); FPRC 5 per mille Ministero della Salute 2022 CARESS (to G.S.); 10.13039/501100003407Ministero dell’Istruzione, dell’Università e della Ricerca (PRIN 2020EK82R5 and P2022C948R) (to G.S.); Università di Torino, Bando Ricerca Locale 2019 (CUP D84I19002940005) (to G.S.); 10.13039/501100000289Cancer Research UK (CRUK) Scotland Institute A31287 Advanced Technology Facilities grant A17196 and 10.13039/501100000289CRUK grant A29800 to S.Z.; and 10.13039/100000002National Institutes of Health grant R01HL131836 (to J.Z.). We thank Alessandro Bosetti and Promega for sharing NanoBiT plasmid constructs. We are grateful to Luca Cevenini and Olympus for providing the LV200 Bioluminescence Imaging System. Lydia Sorokin and Reinhard Fässler are gratefully acknowledged for providing the purified human Lam 511 from placenta and β1 integrin-null MEFs, respectively.

## Author contributions

G.S. conceived the project; G.S., G.V., and J.Z. contributed equally to develop the project; G.S., G.V., N.G., M.G., H.Z., K.H., F.C., S.Z., and J.Z. designed the experiments; G.S., J.Z., S.Z., and F.C. supervised the research; H.Z., K.H., F.C., S.Z., and J.Z. provided key reagents, methods, and technologies; G.V., N.G., M.G., H.Z., and K.H. performed the experiments; all authors analyzed the data and interpreted the results; G.V., N.G., M.G., H.Z., K.H., F.C., S.Z., J.Z., and G.S. wrote the paper; and all authors read and approved the manuscript.

## Declaration of interests

The authors declare no competing interests.

## STAR★Methods

### Key resources table

**Table:** 

REAGENT or RESOURCE	SOURCE	IDENTIFIER
**Antibodies**		

Mouse anti α-tubulin	Sigma	Cat#T5168; Clone: B-5-1-2; RRID:AB_477579
Mouse anti-vinculin	Sigma	Cat#V9131; Clone: hVIN-1; RRID:AB_477629
Mouse anti-TNS3	Sigma	Cat#SAB4200416; Clone: TN-17
Mouse anti-β1 integrin	Bio Legend	Cat#303010; Clone: TS2/16; RRID:AB_314326
Mouse anti-β1 integrin	Abcam	Cat#ab30388; Clone: JB1B; RRID:AB_775736
Rat anti-active β1 integrin	BD Bioscience	Cat#553715; Clone: 9EG7; RRID:AB_395001
Mouse anti-β1 integrin	Sigma	Cat# MAB2247; Clone: 12G10
Mouse anti-inactive β1 integrin	EMD Millipore	Cat#MABT821; Clone: mAb13
Rabbit anti-NanoLuc	Promega	Gift from Promega
Mouse anti-α5 integrin (IP)	BD Pharmingen	Cat#555650; RRID:AB_2233951
Rabbit anti-α5 integrin (WB)	Cell Signaling	Cat#4705S
Mouse anti-α2 integrin (IP/WB)	Santa Cruz	Cat#sc-74466; Clone: C-9; RRID:AB_1124939
Rabbit anti- αv integrin (IP/WB)	Abcam	Cat#ab124968; RRID:AB_11129746
Mouse anti-Talin-1	Abcam	Cat#ab157808; Clone: 8D4; RRID:AB_3076684
Rabbit anti-PJA2	Abcam	Cat#ab272665
Rabbit anti-kindlin-2	Atlas antibodies	Cat#HPA040505; RRID:AB_10673300
Mouse anti-kindlin-3	Invitrogen	Cat#PA5-26932
Rabbit anti-Rap1B	Invitrogen	Cat#PA5-29396; RRID:AB_2546872
Mouse anti-RhoJ	Invitrogen	Cat# MA5-26143; RRID:AB_2725234
Mouse anti-RacGAP1	Santa Cruz	Cat#sc-271110; Clone: A-6; RRID:AB_10611939
Mouse anti-VEGF-B	Santa Cruz	Cat#sc-80442; Clone: J-14I; RRID:AB_1131225
Rabbit anti-GFP antibody	Invitrogen	Cat#A-11122; RRID:AB_221569
Goat HRP-conjugated anti-mouse	Jackson Research	Cat#115-035-003; RRID:AB_10015289
Goat HRP-conjugated anti-rabbit	Santa Cruz	Cat#sc-2054; RRID:AB_631748
Anti-Rat IgG-488	Thermo Fisher	Cat#A21470; RRID:AB_10561519
Anti-Rabbit IgG-555	Thermo Fisher	Cat#A21428; RRID:AB_2535849
Anti-Mouse IgG-647	Thermo Fisher	Cat#A21463; RRID:AB_2535869

**Chemicals, peptides, and recombinant proteins**		

CellTiter-Glo	Promega	G7571
Nano-Glo	Promega	N1110
Fluorescence Mounting Medium	DAKO	S3023
Dithiothreitol (DTT)	Sigma	D0632
Idodacetamide (IAA)	Sigma	I6125
Endopeptidase Lyc	New England Biolabs	Neb#P8101
Acrylamide	National Diagnostics	EC-890
Human Fibronectin	R&D Systems	1918-FN
Human placental collagen type I	Sigma	C774
Human placental laminin 511	Gift by Lydia Sorokin (Institute of Physiological Chemistry and Pathobiochemistry, University of Münster, Münster, Germany)	Previously characterized and validated in Sixt et al.^30^
Human VEGF-B	R&D Systems	751-VE
Poly-D-Lysine	Gibco	A3890401
MK-0429	MedChem Tronica	HY-15102

**Critical commercial assays**		

Strep-Tactin Superflow Plus resin	Qiagen	30004
Ni-NTA resin	Qiagen	88221
TMTpro16plex kit	Thermo Fisher	A44520
TiO2 titansphere beads	Hichrom Limited	5020–75010
ReproSil-Pur-C18-AQ beads	Dr Maisch GmbH	r15.aq
Human 3^rd^ generation druggable genome siRNA library	Qiagen	1027421
Ubiquitination Affinity beads	Cytoskeleton	UBA01B
BCA protein assay reagent	Thermo Fisher	23225
Lipofectamine 2000	Invitrogen	11668027
Oligofectamine	Invitrogen	12252011
EGM-2 Endothelial Cell Growth Medium-2 BulletKit	Lonza	185303
DynaGreen™ CaptureSelect™ Anti-IgG-Fc (Multi-Species) Magnetic Beads	Thermo Fisher	Cat#80108G

**Deposited data**		

Raw data	This paper	Figshare https://doi.org/10.6084/m9.figshare.27842265
Uncropped blots	This paper	Figshare https://doi.org/10.6084/m9.figshare.27842265
RNAseq.bam files	This paper	Figshare https://doi.org/10.6084/m9.figshare.27842265
Proteomic data	This paper	PRIDE: PXD051442

**Experimental models: Cell lines**		

Wild-type mouse embryonic fibroblasts (MEFs)	Gift by Reinhard Fässler (Max Planck Institute of Biochemistry, Martinsried, Germany)	Previously characterized and validated in Böttcher et al.^29^
β1 integrin null (β1^−/−^) mouse embryonic fibroblasts (MEFs)	Gift by Reinhard Fässler (Max Planck Institute of Biochemistry, Martinsried, Germany)	Previously characterized and validated in Böttcher et al.^29^
hTERT-immortalized human aortic endothelial cells (TeloHAECs)	ATCC	CRL-4052
Phoenix	ATCC	CRL-3213
HEK293	ATCC	CRL-1573
Expi293F	ATCC	Previously characterized and validated in Li et al.^74^
β1IAS-KI-TeloHAECs	This paper	N/A

**Oligonucleotides**		

siGENOME siRNA human TLN1 #1: GAAGAUGGUUGGCGGCAUU	Dharmacon	J-012949-05
siGENOME siRNA human TLN1 #2: GUAGAGGACCUGACAACAA	Dharmacon	J-012949-06
siGENOME siRNA human TLN1 #3: UCAAUCAGCUCAUCACUAU	Dharmacon	J-012949-07
siGENOME siRNA human TLN1 #4: GAGAUGAGGAGUCUACUAU	Dharmacon	J-012949-08
siGENOME siRNA human FERMT2 #1: GCCCAGGACUGUAUAGUAA	Dharmacon	L-012753-05
siGENOME siRNA human FERMT2 #2: CUACAUAUUUCUCUCAACA	Dharmacon	L-012753-06
siGENOME siRNA human FERMT2 #3: GAACUGAGUGUCCAUGUGA	Dharmacon	L-012753-07
siGENOME siRNA human FERMT2 #4: AAUGAAAUCUGGCUUCGUU	Dharmacon	L-012753-08
siGENOME siRNA human FERMT3 #1: CCGAAUUGUACACGAGUAU	Dharmacon	J-010677-09
siGENOME siRNA human FERMT3 #2: UGGAGCAGAUCAAUCGCAA	Dharmacon	J-010677-10
siGENOME siRNA human FERMT3 #3: ACUACAGCUUCUUCGAUUU	Dharmacon	J-010677-11
siGENOME siRNA human FERMT3 #4: ACUACAAGAGCCAGGACGA	Dharmacon	J-010677-12
siGENOME siRNA human RAP1B #1: GAACAACUGUGCAUUCUUA	Dharmacon	D-010364-01
siGENOME siRNA human RAP1B #2: CAAUGAUUCUUGUUGGUAA	Dharmacon	D-010364-03
siGENOME siRNA human RAP1B #3: GACCUAGUGCGGCAAAUUA	Dharmacon	D-010364-04
siGENOME siRNA human RAP1B #4: AGUAUAAGCUAGUCGUUCU	Dharmacon	D-010364-05
siGENOME siRNA human TNS3 #1: GGAAAUGACUGAUGCUCGA	Dharmacon	D-009997-21
siGENOME siRNA human TNS3 #2: GGUCCGAACACUUGUACAA	Dharmacon	D-009997-22
siGENOME siRNA human TNS3 #3: GCUCAUUCAUUGUUCGAGA	Dharmacon	D-009997-23
siGENOME siRNA human TNS3 #4: GGACGGAUAAGACGGAAGA	Dharmacon	D-009997-24
siGENOME siRNA human RACGAP1 #1: CAAAUUAUCUCUGAAGUGU	Dharmacon	D-008650-01
siGENOME siRNA human RACGAP1 #2: CCACAGACACCAGAUAUUA	Dharmacon	D-008650-02
siGENOME siRNA human RACGAP1 #3: GAACAUCAGCUUCUCAAGA	Dharmacon	D-008650-03
siGENOME siRNA human RACGAP1 #4: GUAAUCAGGUGGAUGUAGA	Dharmacon	D-008650-04
siGENOME siRNA pool human LPHN2	Dharmacon	Previously characterized and validated in Camillo et al.^38^
siGENOME siRNA pool human FLRT2	Dharmacon	Previously characterized and validated in Camillo et al.^38^
siRNA human pool PLXND1	Sigma	Previously characterized and validated in Rehman et al.^93^
DsiRNA human RHOJ #1: AUGUUUCUACCAAAGCUAUUAGAAC	IDT	hs.Ri.RHOJ.13.1
DsiRNA human RHOJ #2: AGCACACUGCUACUUGGAAUGUUCA	IDT	hs.Ri.RHOJ.13.2
DsiRNA human RHOJ #3: ACCUCUCACUUACGAGCAUGGUGTG	IDT	hs.Ri.RHOJ.13.3
DsiRNA human RHOJ #4: GAGCAUAAAGAUACGUGUUUAAAAA	IDT	hs.Ri.RHOJ.13.4
DsiRNA human PJA2 #1: GCUUUCAUGUUGGAUGGUAACAATA	IDT	hs.Ri.PJA2.13.1
DsiRNA human PJA2 #2: CUUGUGGUUCAGCAUUGAAUCAAAC	IDT	hs.Ri.PJA2.13.2
DsiRNA human PJA2 #3: GUUGAGACAGUGCAUCCAUUUUCTT	IDT	hs.Ri.PJA2.13.3
DsiRNA human PJA2 #4: GUAUUGCAGAAGCACCCUAAACCTT	IDT	hs.Ri.PJA2.13.4
DsiRNA human VEGFB #1: AUGAAUGUCUGCAUCACUAAAUCCA	IDT	hs.Ri.VEGFB.13.1
DsiRNA human VEGFB #2: GGGCAUGAAUGUCUGCAUCACUAAA	IDT	hs.Ri.VEGFB.13.2
DsiRNA human VEGFB #3: CAGUGUGAAUGCAGACCUAAAAAAAA	IDT	hs.Ri.VEGFB.13.3
DsiRNA human VEGFB #4: GAAUGUCUGCAUCACUAAAUCCAGA	IDT	hs.Ri.VEGFB.13.4

**Recombinant DNA**		

Human WT α_5_β_1_ ectodomain with C-terminal ACID-BASE coiled-coil	Li et al.^74^	N/A
pD2529-CAG-acid coiled coil-Integrin α_5_	This paper	N/A
Cas9-2A-EGFP	ATCC	N/A
β1IAS donor plasmid	ATCC	N/A
pCMV6-mPJA2	Origene	MR220203L3
pCMV6	Origene	PS100001
pEGFP-kindlin-2	Gift by Reinhard Fässler (Max Planck Institute of Biochemistry, Martinsried, Germany)	N/A
pEGFP-N1	Addgene	Cat#6085-1

**Software and algorithms**		

PRISM (10.1.0) GraphPad	GraphPad	https://www.graphpad.com/scientific-software/prism/
Fiji (ImageJ)	NIH	https://imagej.net/software/ fiji/downloads
Leica Application Suite	Leica	https://www.leica-microsystems.com/products/microscope-software/p/leica-application-suite/
Summit 4.3 software	This paper	N/A
Real-Time Cell Analyzer (RTCA) software	ACEA Biosciences/Agilent Technologies	https://www.agilent.com/en/product/cell-analysis/real-time-cell-analysis/rtca-software/software-download
RCSB Protein DataBank	NIH	https://www.rcsb.org/
Xcalibur software	Thermo Fisher	OPTON-30965
MaxQuant (software^94^ version 1.6.14)	Cox and Mann^94^	N/A
Andromeda search engine	Cox et al.^95^	N/A
Perseus (software version 1.6.15.0. Reverse)	Tyanova et al.^96^	N/A
EnrichR package	This paper	N/A

### Experimental model and study participant details

#### Cell line and culture

Wild-type and β1^−/−^ integrin Mouse Embryonic Fibroblasts (MEFs) were a kind gift from Reinhard Fässler (Max Planck Institute of Biochemistry, Martinsried, Germany) and cultured in DMEM completed with glutamine, penicillin/streptomycin solution (Sigma-Aldrich) and 10% Fetal Bovine Serum (Euroclone). Immortalized Telo Human Aortic Endothelial Cells (TeloHAECs) from American Tissue Cell Culture (ATCC) were used (CRL-4052) and grown with EGM-2 Endothelial Cell Growth Medium-2 BulletKit (Lonza). When poor medium is used, this corresponds to EGM-2 without vascular endothelial growth factor-A (VEGF-A) or other growth factors contained in the BulletKit. To measure cell response to different matrix stiffness, MEF or TeloHAEC cells were plated on human fibronectin, 1918-FN (R&D Systems)-coated PAA gels (6% [0.5, 10 or 100 kPa] of gel diluted from 30% Protogel in phosphate buffered saline (PBS), 37.5:1 fixed ratio of acrylamide:bis-acrylamide; EC-890, National Diagnostics), previously prepared in glass slide with removable silicon chamber (IBIDI), according to published methods, with modifications.^38^ Phoenix cells were purchased from ATCC (CRL-3213) and cultured in DMEM completed with glutamine, penicillin/streptomycin solution (Sigma-Aldrich) and 10% Fetal Bovine Serum (Euroclone).

#### Generation of β1IAS KI ECs

The CRISPR-Cas9 technology knock-in was performed in collaboration with American Tissue Cell Culture (ATCC) and it was exploited to insert the LgBiT (ATGGTCTTCACACTCGAAGATTTCGTTGGGGACTGGGAACAGACAGCCGCCTACAACCTGGACCAAGTCCTTGAACAGGGAGGTGTGTCCAGTTTGCTGCAGAATCTCGCCGTGTCCGTAACTCCGATCCAAAGGATTGTCCGGAGCGGTGAAAATGCCCTGAAGATCGACATCCATGTCATCATCCCGTATGAAGGTCTGAGCGCCGACCAAATGGCCCAGATCGAAGAGGTGTTTAAGGTGGTGTACCCTGTGGATGATCATCACTTTAAGGTGATCCTGCCCTATGGCACACTGGTAATCGACGGGGTTACGCCGAACATGCTGAACTATTTCGGACGGCCGTATGAAGGCATCGCCGTGTTCGACGGCAAAAAGATCACTGTAACAGGGACCCTGTGGAACGGCAACAAAATTATCGACGAGCGCCTGATCACCCCCGACGGCTCCATGCTGTTCCGAGTAACCATCAACAGT) and the SmBiT (GTGACCGGCTACCGGCTGTTCGAGGAGATTCTG) cDNAs, separated by a 15 amino acid long Gly-Ser linker, into the primary sequence (exon 4) of human β1 integrin (ITGB1) gene, corresponding to the S_79_-K_85_ loop of the ternary structure, as shown in Figure 1A.

#### gRNA design

Three gRNAs (one binding to the sense strand and the other two to the antisense one) were selected to be less than 15 nucleotides (nt) apart from the target insertion site and predicted to have low off-targets. gRNA #1 sequence: AATGTAACCAACCGTAGCAAAGG, gRNA #2: TGCTGTTCCTTTGCTACGGTTGG and gRNA #3: TCTCTGCTGTTCCTTTGCTACGG. Each gRNA was cloned into Cas9-2A-EGFP vector and Neon transfected together with the donor plasmid into TeloHAEC cells.

#### Donor design

The donor was designed so that the plasmid DNA contains the Gly-Ser linker separated LgBiT and the SmBiT sequences (555 bp) inserted into the target site of exon 4 of human β1 integrin gene, flanked by a 800 bp Left Homology Arm (LHA) and a 800 bp Right Homology Arm (RHD).

The selected gRNA (g1) into the Cas9-2A-EGFP vector was Neon transfected with the donor plasmid into parental wild type TeloHAECs that were then single clone selected by GFP sorting and limiting dilution. After clone expansion, genomic DNA was extracted and sequence was confirmed by Sanger sequencing.

### Method details

#### Recombinant proteins and luminescence assay

Human wild type α_5_β_1_ ectodomain with C-terminal ACID-BASE coiled-coil, Strep-Tag II, and His-tag was expressed in HEK293 cells as described before.^74^ The protein was purified using Strep-Tactin Superflow Plus resin (Qiagen) and gel filtrated using Superdex 200 Increase 10/300 GL (Cytiva). For the construct of α_5_β_1_ IAS ectodomain, integrin α_5_ was cloned into pD2529-CAG-acid coiled coil vector with Strep-Tag II, β1IAS was cloned into pD2529-CAG-basic coiled coil vector with His-tag. An HRV 3C protease cleave site was present before the coiled coil sequence in both subunits. The protein was expressed in Expi293F cells and purified using Ni-NTA resin (Qiagen), and then concentrated and buffer exchanged in TBS buffer (25 mM Tris pH 8.0, 150 mM NaCl, 1mM Ca^2+^, 1mM Mg^2+^). Western blot was used to confirm the presence of α5β1IAS. Wild type α5β1 ectodomain was used as control. Purified unclasped α5β1IAS or wild type α5β1 integrin ectodomains were generated by incubating their clasped counterparts overnight at room temperature with HRV 3C protease, in a 1:100 protease to integrin weight ratio.

200 μL of TBS buffer (Ctl), TBS buffer with 5% BSA, or TBS buffer with 5 μg/mL FN was added into each well of 96 well plate and incubated overnight at 4°C. The wells were washed three times with TBS buffer. 50 μL of the purified α5β1IAS ectodomain treated with or without HRV 3C protease in TBS buffer containing either 1 mM Ca^2+/^Mg^+^ or additional 2 mM Mn^2+^ was added into each well and incubated for 20 min. 50 μL of NanoBiT Luciferase Assay Substrate (Promega) was added into each well and incubated for 5 min. The luminescence intensity was measurement using EnVision 2105 multimode plate reader (PerkinElmer).

#### Luminescence assay in living cells

For experiments on increasing FN, Coll I, Lam 511 amounts and stiffness, solid-white flat 96-well plate was coated with 500 ng/mL FN for 1 h at 37°C and then saturated with 3% BSA in PBS for 1h at 37°C. Next, 8 × 10^3^ cells/well (MEFs or ECs) were plated in triplicates and let adhere for 30 min. For experiments with 9EG7 (10 μg/mL), 12G10 (10 μg/mL), mAb13 (10 μg/mL) and MK-0429 (100 μM), cells were given the treatment, while adhering for 15 min in complete medium. For experiments with VEGF-B, cells were added to each well in presence or not with 200 ng/mL of VEGF-B in poor medium for 15 min. Next, NanoBiT Luciferase Assay Substrate (Promega) was added 1:80 in OptiMEM to each well. After 10 min (included in the adhesion time) incubation at 37°C, the luminescent signal was detected with the Spark multimode microplate reader (Spark). For clarity, the 10 min of incubation with the NanoBiT Luciferase Assay Substrate was always included in the final adhesion timing tested.

#### siRNA-based high throughput screening (HTS)

The siRNA HTS was performed in collaboration with Misvik Biology (Turku, Finland) and the human 3rd generation druggable genome siRNA library (Qiagen), containing 6948 targeting siRNAs (pool of 4 individual siRNAs per gene) together with 1148 control siRNAs was used. Different types of control siRNAs were randomly distributed in the library: GFP, Scrambled, and AllStars negative control. For the HTS experiments, 24x384-well plates were coated with 500 ng/mL FN for 1 h at 37°C and then saturated with 3% BSA in PBS for 1h at 37°C. The Qiagen pooled siRNA library readily diluted in Qiagen siRNA buffer was transferred to the FN-coated wells in a final siRNA concentration of 64 nM per well, using a 96-channel liquid handling robot (Eppendorf EpMotion). Raiman lipid transfection reagent was added to the wells according to manufacturer’s protocols (Invitrogen) and incubated for 20 min at room temperature. 6× 10^2^ β1IAS KI ECs in complete medium was dispensed in each 384-well plate well and allowed to sediment for 20 min at room temperature. Cells were transfected in complete medium for 72h and then incubated with NanoBiT Luciferase Assay Substrate 1:80 in HBSS buffer for 10 min at 37°C using a Multidrop Combi (Thermo Fisher Scientific) liquid dispenser, and the luminescent signal was detected with Labrox multimode multiplate reader (Uniogen).

#### HTS validation

For HTS candidate validation experiments, β1IAS KI ECs were seeded, in quadruplicates, in solid-white flat 384-well plate coated with 500 ng/mL FN at a concentration of 2 × 10^3^ cells/well, the day before oligofection. Oligofection of siRNA duplexes was performed according to manufacturer’s protocols (Invitrogen). Briefly, cells were transfected twice (at 0 and 24 h) with 200 pmol of siGENOME Non-Targeting siRNA #1 D-001206-13 as control (siCTL) or siGENOME SMART pools (pool of 4 individual siRNAs per gene) (Dharmacon) for human TLN1, FERMT2, FERMT3, FLRT2, LPHN2, PLXND1, RAP1B, TNS3, RACGAP1 or four siRNA pools (IDT) for human RHOJ, PJA2, VEGFB, in a final siRNA concentration of 200 nM. 24, 48 or 72 h after the second oligofection, cells were incubated with NanoBiT Luciferase Assay Substrate (Promega) 1:80 in OptiMEM for 10 min at 37°C, and the luminescent signal was detected with Spark multimode microplate reader (Spark). The effect of each gene silencing on cell viability was assessed using CellTiter-Fluor Cell Viability Assay (Promega).

#### MS proteomic analysis

Parental wild type TeloHAECs were plated on 500 ng/mL of FN and let to adhere in presence or not of 200 ng/mL of human VEGF-B 167 (751-VE, R&D System) for 15 or 30 min, in poor medium. Cells were lysed in Chromatin H_2_0, 2.5% sodium dodecyl sulfate (SDS), 250 mM Tris-HCl, pH 6.8, at 95°C. 100 μg of sample from each condition was reduced, alkylated and digested overnight with 5 mM final concentration dithiothreitol (DTT, Sigma), 55 mM idodacetamide (IAA, Sigma), 1:40 Endopeptidase Lyc (New England Biolabs Inc) then 1:100 enzyme to protein trypsin (Promega), respectively. Digested samples were then labeled with 1 x TMTpro16plex kit (Thermo Fisher Scientific) before pooling to a single sample. Phosphorylated peptide enrichment was performed on the pooled sample. TiO2 titansphere (Hichrom Limited) beads were prepared at a 1:5 sample:bead ratio and activated with 80% ACN:6% TFA and slurry placed into a 200 μL tip with a C18 empore disk (3M) stopper. Sample was loaded onto tip and spun slowly through the packed tip at ∼500 g. This was repeated x 3 before the flow through was collected and loaded onto a fresh tip/slurry mix for a second round of enrichment. Bound phosphorylated peptides were washed with 100 μL 30% ACN:6% TFA then 3 x 100 μL 80% ACN:0.3% TFA before elution with 40 μL 15% NH4OH:40% ACN. Samples were then dried down to complete dryness before fractionation.

Enriched phosphorylated peptides samples were fractionated into 8 fractions using high pH on tip fractionation, utilizing a mix of buffer A, 200 μM ammonium formate pH10, and buffer B, 100% ACN spun through slurry at 1000 g for 1min between each step. Briefly, 50 μL of 3 mg ReproSil-Pur-C18-AQ 5 μm (Dr Maisch GmbH) beads were loaded onto a 200μL tip with a C18 empore disk stopper and allowed to settle for 5 min 50 μL of 100% MeOH was added to activate beads then equilibrated with 200 μM ammonium formate pH10: 50% ACN, then 200μM ammonium formate pH10: 12.5% ACN. 50 μL of 200 μM ammonium formate pH10 was loaded. Sample was re-suspended in 100 μL 200 μM ammonium formate pH10, loaded onto tip and soaked for 10 min before sample loaded. Elution was performed using an increasing % of ACN from fraction 1 to 8: 5%, 7.5%, 10%, 12.5%, 15%, 17.5%, 20% and 50% respectively.

Peptide samples from each of the 8 fractions were run on an Orbitrap Lumos mass spectrometer (Thermo Scientific) coupled to an EASY-nLC II 1200 chromatography system (Thermo Scientific). Samples were loaded onto a 50 cm fused silica emitter (packed in-house with ReproSIL-Pur Basic C18, 1.9 μm resin, Dr Maisch) which was heated to 50°C using a column oven (Sonation). Samples were run with solvents A (0.1% FA) and B (80% ACN, 0.1% FA). Samples were eluted at a flow rate of 300 nL/min over 8 optimized two-step gradient methods. Step one was commenced for 75 min and step two for 25 min. For fraction 1 the % of solvent B was 2–16% at step one and 27% at step two, 3–17% then 30%, 3–19% then 33%, 4–20% then 33%, 4–23% then 37%, 7–26% then 43%, 8–33% then 47%, 9–37% then 53% for fractions 2–8 respectively. Peptides were electrosprayed into the mass spectrometer using a nanoelectrospray ion source (Thermo Scientific). An Active Background Ion Reduction Device (ESI Solutions) was used to decrease air contaminants.

Data was acquired with Xcalibur software (Thermo Scientific) in positive ion detection mode utilizing data dependent acquisition. Full scan mass (MS1) range was set to 375–1400 m/z at 60,000 resolution. Injection time was set to 50 ms with a target value of 3E4 ions. HCD fragmentation was triggered for the top15 most intense ions for MS2 analysis. MS2 injection time was set to 120 ms with a target of 200% AGC, 0.8m/z isolation window and resolution of 50,000. Ions that had already been selected for MS2 were dynamically excluded for 30 s.

#### MS data analysis

Data was processed using MaxQuant software^94^ version 1.6.14 and searched with the Andromeda search engine^95^ against the Uniprot Homo Sapiens database (Swiss-prot, 20,043 entries, 2023). Data was searched with multiplicity set to MS2 level TMT16plex. MS/MS mass tolerance was set to 20 ppm and minimum peptide length was 7 amino acids. Trypsin was selected as the digestion enzyme allowing for 2 missed cleavages and methionine oxidation and N-terminal acetylation and Phospho (STY) was set as variable modifications. Carbamidomethyl was set as a fixed modification. False discovery rate was set to 1%. MaxQuant output was processed using Perseus software^96^ version 1.6.15.0. Reverse and Contaminant Phosphorylation sites were removed and the sites filtered based on a score difference greater than 5 and a localization probability greater than 0.5. Data was normalized by median and logged (log2). For comparative analysis, standard Student’s T test was applied and sites with a difference of ≥0.2 and a *p*-value ≤0.05 were considered significant. Gene Ontology Biological Process annotations from UniProt curated database and kinase motifs, kinase-substrate interactions, regulatory sites and known sites were added from PhosphoSite Plus^97^ v6.7.1.1 to Table S3.

#### Microscopy

##### Confocal microscopy

Cells, previously fixed for 10 min in PBS 2% paraformaldehyde (PFA) on wet ice, were permeabilized with Triton 0.1% in PBS for 2 min at 4°C. Primary antibodies were diluted in PBS 1% donkey serum for 1h at room temperature and revealed by appropriate secondary antibodies labeled with fluorochromes (Alexa , Invitrogen), diluted in PBS 1% donkey serum 1:100 for 30 min at room temperature. Only for 9EG7 active integrin staining, the primary antibody was given to live cells (10 μg/mL in complete medium) for 15 min and then PFA fixed, permeabilized and stained as described. The slides were mounted on a microscope slide using Fluorescence Mounting Medium (DAKO) and were allowed to dry overnight at room temperature. The cells were observed under a confocal microscope (TCS SP8 AOBS, Leica Microsystems, Mannheim, Germany). For image acquisition we used a 63× oil-immersion objective (N.A. 1.32). Image acquisition was performed by adopting the same laser power, gain, and offset settings for all the images of the same experiment and avoided saturation.

Images were analyzed using the Leica Application Suite (for colocalization analysis) or ImageJ quantification tool (for relative mFeret Diameter, mFD).

##### Luminescence microscopy

Wild-type or transduced cells with β1IAS or NanoLuc alone were plated on fibronectin (3 μg/mL) and let adhere in a glass bottom μ-slide 8-well (ibidi) for 3 h. 10 min before imaging, cells well incubated with NanoBiT Luciferase Assay Substrate (Promega) 1:80 in OptiMEM at 37°C. The luminescent signal was observed under a luminescent microscope (Luminoview, LV-200 MD, Olympus, Shinjuku, Japan). For image acquisition we used a 40× oil-immersion objective (N.A. 1.30). Image acquisition was performed by adopting the same laser power, gain, and offset settings for all the images of the same experiment and avoided saturation.

##### Flow cytometry analysis

Cells were trypsinized, aliquoted (0.5 × 10^6^ cells per condition) and washed with 1% BSA in PBS (FACS buffer). Then, the cells were resuspended and incubated with the primary antibody (30′ on ice) on oversaturating concentration (2μg). Unbound primary antibody was washed away by washing in FACS buffer and the resuspended cells were incubated with 1 μg Alexa Fluor 488-conjugated Donkey anti-rat for 30 min. After a wash in FACS buffer, cells were resuspended in 500μL of PBS.

All samples were acquired using Summit 4.3 software on a Dako CyAN ADP flow cytometer.

#### Western blot analysis

Cells were warm lysed in H_2_0 buffer containing 25% Sodium Dodecyl Sulfate (SDS), 25 mM Tris-HCl, 100 mM NaCl, pH 7.6 at 95°C. The total protein amount was determined using the bicinchoninic acid (BCA) protein assay reagent (Thermo Fisher Scientific). Equivalent amount of protein (20–30 μg) was separated by SDS– polyacrylamide gel electrophoresis (PAGE) with precast Bolt 4–12% Bis-Tris gel (Thermo Fisher Scientific). Proteins were then transferred to a nitrocellulose membrane (Bio-Rad), blocked in 5% Bovine serum albumin (BSA) buffer for 1h at RT, incubated with appropriate primary antibodies in PBS-Tween 0.1%, overnight at 4°C or 1h at RT. The HRP-conjugated secondary antibodies were detected by enhanced chemiluminescence technique (BioRad).

#### Immunoprecipitation

To immunoprecipitate and analyze by Western blot β1IAS dimerization with other α integrin subunits, β1IAS ECs were washed 3 times with PBS and lysed with a buffer containing 25 mM Tris–HCl pH 7.6, 100 mM NaCl, 0.15% Tween 20, 5% glycerol, 0.5 mM EGTA, 2 mM MgCl_2_, 1 mM PMSF, 1 mM Na_3_VO_4_, and protease inhibitor cocktail (Sigma Aldrich). Cellular lysates were incubated for 20 min on ice and then centrifuged at 15,000 *g*, 20 min, at 4°C. The total protein amount was determined using the bicinchoninic acid (BCA) protein assay reagent (Thermo Fisher Scientific). Equivalent amount (1 mg) of protein were precipitated for 1 h at 4°C with DynaGreen CaptureSelect Anti-IgG-Fc (Multi-Species) Magnetic Beads (Thermo Fisher Scientific) and then centrifuged for 1 min 15,000 *g* at 4°C. DynaGreen Magnetic beads were collected and incubated with the anti-α5, α2 or αv antibodies for 1 h at 4°C. Immunoprecipitates were washed four times with lysis buffer and then separated by SDS–PAGE. Proteins were then transferred to a nitrocellulose membrane (Bio-Rad), probed with NanoBiT antibody, and detected by enhanced chemiluminescence technique (PerkinElmer).

#### Ubiquitin assay

Pja2 and kindlin-2 levels were co-overexpressed in Phoenix cells by Lipofectamine 2000 transfection reagent (Invitrogen) in OptiMEM, for 5 h at 37°C. Cells were also co-transfected with kindlin-2 together with a control vector. After overnight growth in standard conditions, cells were lysed as described above. 1.5 mg of each lysate was incubated on 20 μL of reconstituted UBA01B-beads (Cytoskeleton) for 2 h at 4°C. Moreover, as described by the bead manufacturer, 20 μL of reconstituted control beads (Cytoskeleton) were incubated with lysate of Phoenix cells co-transfected with both control vectors in the same conditions. Protein run was performed as previously described and ubiquitinated kindlin-2 levels were detected with GFP antibody (Invitrogen, A-11122).

#### Cell spreading assay

Real-time adhesion of MEFs or ECs was monitored with an xCELLigence, an electrical impedance-based system in which microelectronic sensor arrays are integrated into the bottom of microplate wells, allowing cells in the wells to be constantly monitored. The xCELLigence system is based on the Real-Time Cell Analyzer (RTCA) instrument (ACEA Biosciences/Agilent Technologies). Its software converts impedance values to obtain parameters such as cell index (CI), mean values, and standard deviation (SD). In detail, the bottom side of the E-Plate 16 was coated with 1.5 μg/mL FN for 1 h at room temperature. Then, the protein-coated plate was washed with PBS and incubated with 3% BSA solution in PBS for 1 h at 37°C. Cells were detached by means of trypsin-EDTA and resuspended to a final concentration of 8000 cells/100 μL. The BLANK step was started to measure the background impedance of cell culture medium, which was then used as reference impedance for calculating CI values. 100 μL of cell suspension (8000 cells) was then added to each well. For spreading assay with VEGF-B, cells were added to each well in presence or not with 100 or 200 ng/mL of VEGF-B in poor medium. The E-Plate 16 was placed in the RTCA DP Instrument equilibrated in a CO2 incubator. Cells adhesion was continuously monitored using the RTCA DP instrument. Mean, SD, and *p*-value were calculated on the CI data, exported from the RTCA instrument, including the technical replicates of each experimental condition at each time-point.

#### RNA sequencing

RNA sequencing was performed on β1IAS KI ECs in three independent experiments by Misvik Biology. The RNA libraries were sequenced on a paired-end read flow cell by bridge amplification and 150 cycles sequencing-by- synthesis on the Illumina NovaSeq 6000 S4 platform (Illumina Inc., San Diego, CA, USA). The average number of raw reads obtained was 39.3 million.

#### Bioinformatics analysis

The RNA-sequencing (RNA-seq) reads were aligned to the reference genome v hg38/GRC38 using Bowtie v 2.3.3.1, the annotation was performed on Gencode v29. Read count and normalization analysis was performed with DESeq2 v1.38.3. A transcript is considered in the analysis if associated with a median value of reads >20.

Enrichment analysis was performed on 194 candidate genes selected setting using a *Z* score threshold equals to 2 standard deviations. EnrichR web-based tool was used to combine KEGG, WikiPathways, NCI-Nature, BioPlanet databases. Only terms associated with an adjusted *p*-value <0.05 were considered as significant. Among the significant pathways, those related to integrin function and to cell adhesion were chosen for representation.

### Quantification and statistical analysis

For statistical evaluation, data distribution was assumed to be normal. Parametric two-tailed heteroscedastic Student’s t-test was used to assess the statistical significance when two groups of unpaired normally distributed values were compared; when more than two groups were compared, parametric two-tailed analysis of variance (ANOVA) with Bonferroni’s correction was applied. Selected *p* values are shown as absolute values and all *p* values are included in Table S1.

For each experiment, we performed 2–5 independent biological replicates, in which at least three or four technical replicates were performed. Then, to analyze the reproducibility, we calculated the mean of the independent biological replicates ±SD and performed statistical analyses.

For HTS data analysis, normalization of the detected luminescent raw signals was performed plate by plate based on GFP, Scrambled and AllStars negative controls. *Z* score was calculated as $\frac{\chi -\mu }{\sigma }$ of the luminescent emission signal data (χ = data point, μ = mean, σ = standard deviation).

## Supplemental information

Document S1. Figures S1–S4

Table S1. *p* values for ANOVA comparisons

Table S2. HTS significant pathways

Table S3. MS proteomics analysis data

Document S2. Article plus supplemental information
