# Supplementary material for: Luminescent sensing of conformational integrin activation in living cells
Source: Cell Rep. 2025 Feb 17;44(2):115319. doi: 10.1016/j.celrep.2025.115319 (PMC11861568; doi:10.1016/j.celrep.2025.115319)
Supplement: Document S1. Figures S1–S4 [file mmc1.pdf]

Cell Reports, Volume *44*

Supplemental information

Luminescent sensing of conformational integrin activation in living cells

Giulia Villari, Noemi Gioelli, Marta Gino, Heng Zhang, Kelly Hodge, Francesca Cordero, Sara Zanivan, Jieqing Zhu, and Guido Serini

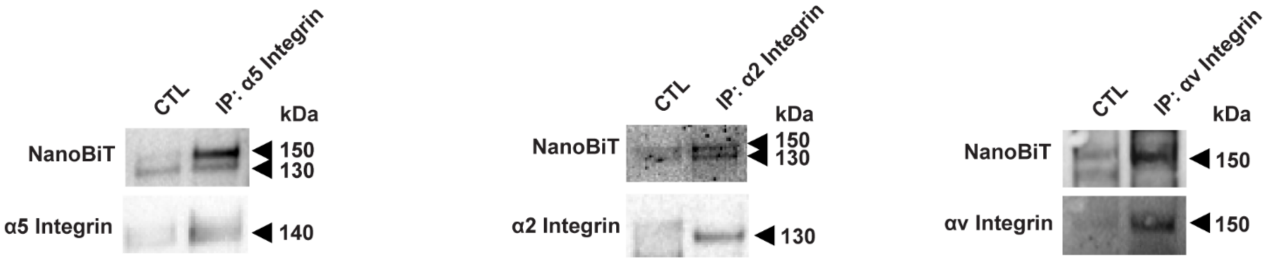

Figure S1. β1IAS interacts with different integrin α subunits, related to Figure 3. Western blot showing β1IAS immunoprecipitated with α5 (left), α2 (middle) and αv (right) integrins in β1IAS KI ECs, compared to CTL.

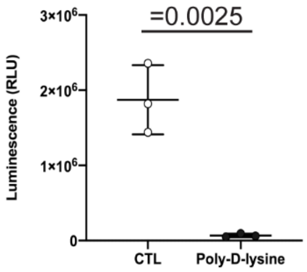

Figure S2. Luminescence emitted by β1IAS ECs plated on poly-D-lysine compared to fibronectin, related to Figure 3. Measurement of luminescence intensity emitted by β1IAS KI ECs adhering for 30 minutes on poly-D-lysine (1 mg/ml), compared to that emitted by the same cells on FN as a control (CTL). Data are mean ± SD of three independent experiments. Statistical analysis: two-tailed heteroscedastic Student’s t-test.

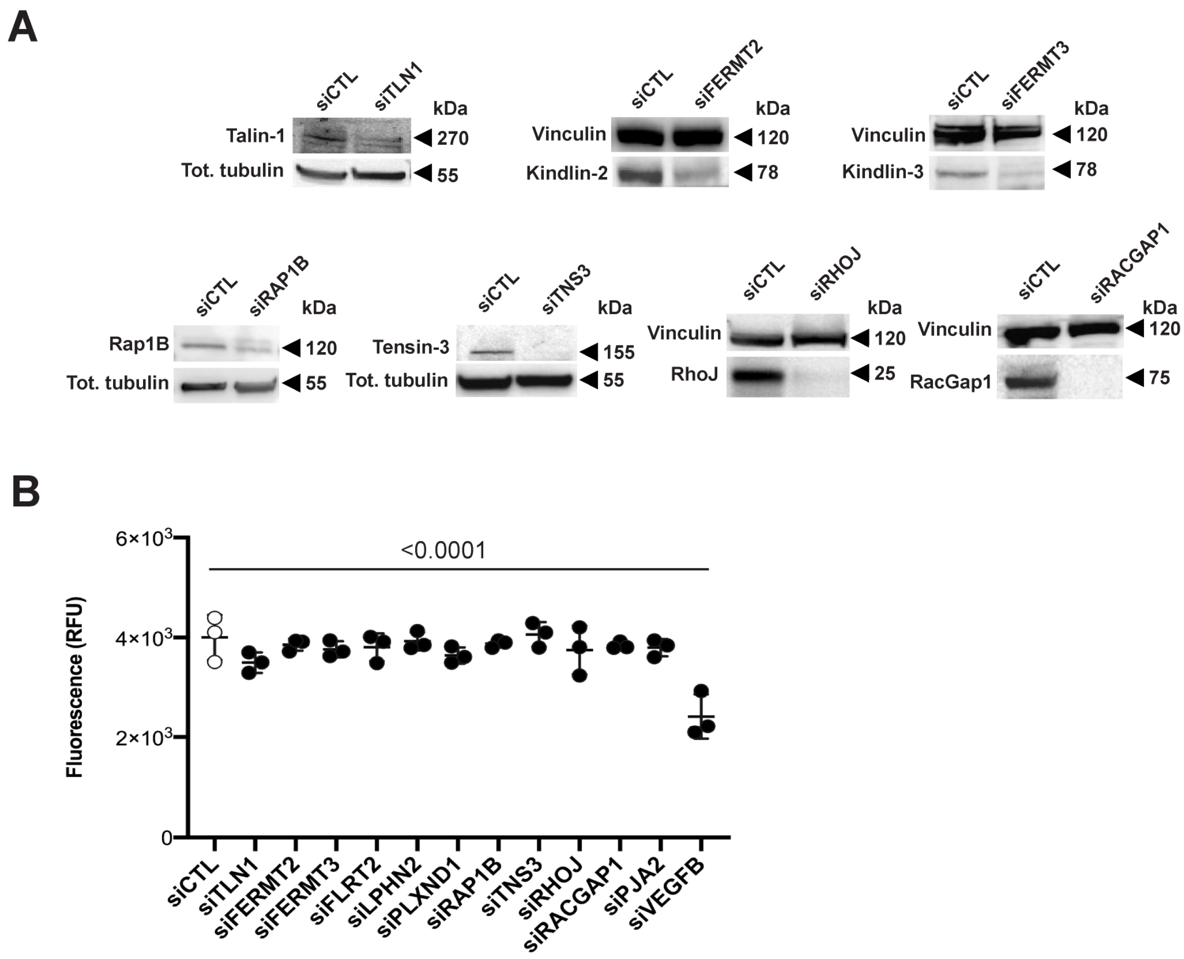

Figure S3. Expression levels of β1 integrin activators and inhibitors, and analysis of cell viability upon silencing in β1IAS ECs, related to Figure 4. (**A**) Western blot analyses upon silencing of TLN1, FERMT2, FERMT3, RAP1B, TNS3, RHOJ and RACGAP1 in β1IAS KI ECs, compared to siCTL. (**B**) Cell viability in β1IAS KI ECs silenced for TLN1, FERMT2, FERMT3, FLRT2, LPHN2, PLXND1, RAP1B, TNS3, RHOJ, RACGAP1, PJA2 and VEGFB. Data are the mean ± SD of three independent experiments. Statistical analysis: two-way ANOVA and Bonferroni's post hoc analysis.

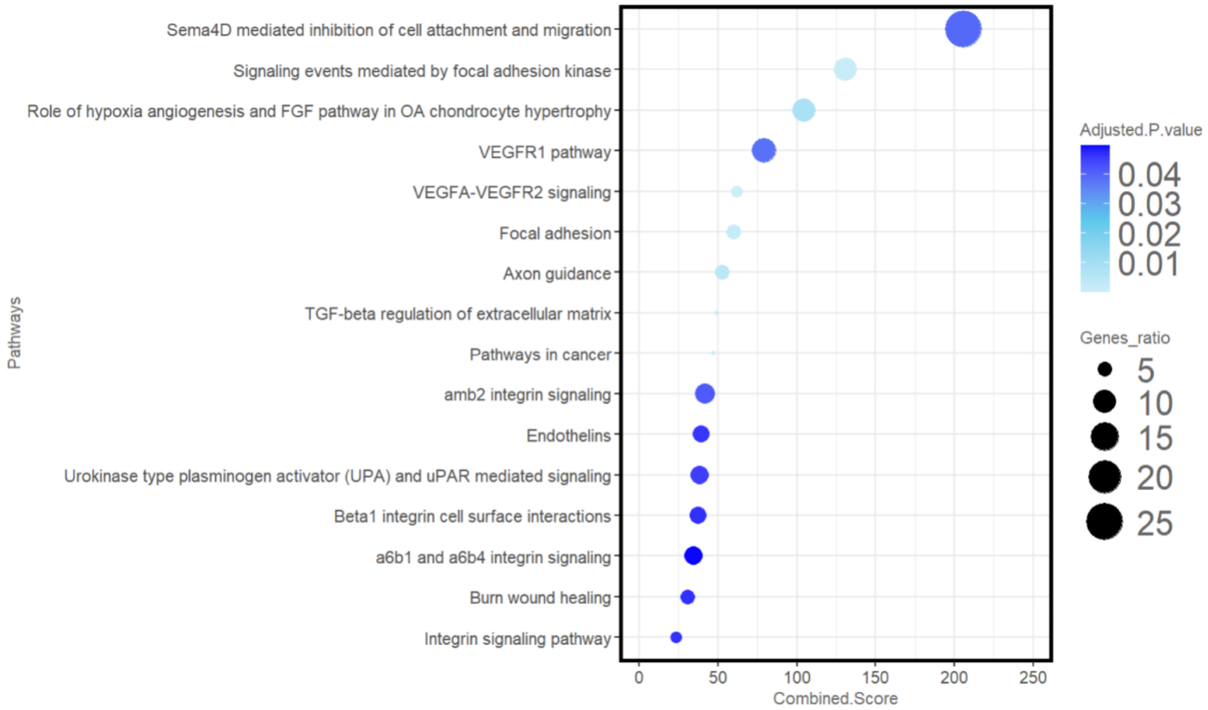

Figure S4. Bioinformatic identification of top enriched pathways emerging from siRNA HTS on β1IAS KI ECs, related to Figure 4. Bubble plot representing top cell adhesion related enriched pathways (adjusted p-value <0,05) based on candidate genes obtained from the HTS (2 <Z-score< -2). EnrichR was used to combine KEGG, WikiPathway, NCI-Nature, BioPlanet databases. The combined score is the log of the p-value from the Fisher exact test multiplied by the z-score of the deviation from the expected rank. Bubble color (adjusted p-value) was computed using the Benjamini-Hochberg method for correction for multiple hypotheses testing. The gene ratio is the overlap between the input list and the gene sets in each gene-set library for ranking a pathway's relevance to the input list.
